# Translationally controlled tumor protein (TCTP) plays a pivotal role in cardiomyocyte survival through a Bnip3-dependent mechanism

**DOI:** 10.1038/s41419-019-1787-7

**Published:** 2019-07-18

**Authors:** Wenqian Cai, Takayuki Fujita, Yuko Hidaka, Huiling Jin, Kenji Suita, Mayo Shigeta, Hiroshi Kiyonari, Masanari Umemura, Utako Yokoyama, Junichi Sadoshima, Yoshihiro Ishikawa

**Affiliations:** 1 Cardiovascular Research Institute, Yokohama City University Graduate School of Medicine, Yokohama, Japan; 2 Laboratories for Animal Resource Development, RIKEN Center for Biosystems Dynamics Research, Kobe, Japan; 3 Laboratories for Genetic Engineering, RIKEN Center for Biosystems Dynamics Research, Kobe, Japan; 4 Department of Cell Biology and Molecular Medicine, Cardiovascular Research Institute, Rutgers New Jersey Medical School, Newark, NJ USA

**Keywords:** Cell death, Heart failure

## Abstract

Prevention of cardiomyocyte death is an important therapeutic strategy for heart failure. In this study, we focused on translationally controlled tumor protein (TCTP), a highly conserved protein that is expressed ubiquitously in mammalian tissues, including heart. TCTP plays pivotal roles in survival of certain cell types, but its function in cardiomyocytes has not been examined. We aimed to clarify the role of TCTP in cardiomyocyte survival and the underlying mechanism. Here, we demonstrated that downregulation of TCTP with siRNA induced cell death of cardiomyocytes with apoptotic and autophagic features, accompanied with mitochondrial permeability transition pore (mPTP) opening. TCTP loss did not induce cell death of cardiac fibroblasts. Bcl-2/adenovirus E1B 19-kDa interacting protein 3 (Bnip3) was found to mediate the TCTP-loss-induced cardiomyocyte death. In exploring the clinical significance of the TCTP expression in the heart, we found that DOX treatment markedly downregulated the protein expression of TCTP in cultured cardiomyocytes and in mouse heart tissue. Exogenous rescue of TCTP expression attenuated DOX-induced cardiomyocyte death. In mice, cardiomyocyte-specific overexpression of TCTP resulted in decreased susceptibility to DOX-induced cardiac dysfunction, accompanied with attenuated induction of Bnip3. Dihydroartemisinin, a pharmacological TCTP inhibitor, induced development of heart failure and cardiomyocyte death in control mice, but not in mice with cardiomyocyte-specific TCTP overexpression. Our findings revealed TCTP has a pivotal role in cardiomyocyte survival, at least in part through a Bnip3-dependent mechanism. TCTP could be considered as a candidate therapeutic target to prevent DOX-induced heart failure.

## Introduction

Prevention of cardiomyocyte death is an important therapeutic strategy for heart failure^1,2^. The loss of cardiomyocytes induced by various stresses is a major cause of reduced cardiac performance. Numerous studies have revealed the involvement of several pathways, including catecholamine and p53-mediated signaling, in stress-induced cardiomyocyte death and subsequent development of heart failure^2–11^. However, the major signaling pathway involved in the survival of cardiomyocytes has not been established.

In this study, we focused on translationally controlled tumor protein (TCTP), a highly conserved protein that is expressed ubiquitously in mammalian tissues including heart^12,13^. Recent reports, including our own, have revealed that TCTP plays important roles in various cellular functions including cell survival, cell proliferation, tumorigenesis, allergic response, and pulmonary vascular remodeling^14–18^. However, to our knowledge, there is no report on TCTP function in cardiomyocytes.

TCTP exerts its functions in a cell-type-dependent manner. It promotes cell survival and inhibits apoptosis in some types of normal cells^12,19^ and cancer cells^15,20^. Several studies have demonstrated that TCTP deficiency results in early embryonic lethality characterized by increased apoptosis^12,21^. In addition, TCTP silencing caused induction of DNA damage or apoptosis in several normal^19^ and cancer cell lines^22–24^. These findings indicate that TCTP is a pro-survival molecule. However, the significance of TCTP in cell survival depends on the cell type and conditions. On the other hand, TCTP deficiency does not affect apoptotic sensitivity or proliferation of mouse embryonic fibroblasts^21^. The percentage of TUNEL-positive cells in embryos at day 5.5 showed no difference between wild-type and TCTP-deficient mice^21^. TCTP deletion in T cells caused no reduction in thymocyte numbers^25^. In addition, an inverse relationship between TCTP expression and growth rate was found with an epithelial cell line^26^.

In this study, we examined the role of TCTP in cardiomyocyte survival. We found that TCTP downregulation induced cardiomyocyte death, indicating that TCTP is a key player in cardiomyocyte survival. In addition, we identified Bcl-2/adenovirus E1B 19-kDa interacting protein 3 (Bnip3) as a mediator of TCTP-loss-induced cell death. Further, TCTP-loss-induced cardiomyocyte death had apoptotic and autophagic features accompanied with mPTP opening, which are features of Bnip3-induced cell death^27–29^. TCTP and Bnip3 are both expressed in mitochondria^30^. Recent studies have demonstrated that Bnip3 is involved in cardiomyocyte death in response to clinically important pathogenic stresses, including treatment with anthracycline antibiotics^27,28,31,32^.

In exploring the clinical significance of TCTP expression in the heart, we found that doxorubicin (DOX) treatment markedly suppressed the protein expression of TCTP in cultured cardiomyocytes and mouse heart tissue. DOX is an anthracycline antibiotic, and is widely used in cancer therapy. However, its clinical usage has been limited by its serious cardiotoxicity. Approximately 10% of patients are reported to suffer cardiac side effects^33^, which not only limit their activities of daily life, but also require dose reduction, reducing the effectiveness of treatment for malignancies. Thus, there is an urgent need for a strategy to prevent DOX-induced cardiac dysfunction.

Based on the above findings, we hypothesized that the maintenance of TCTP expression level could be an effective strategy to prevent DOX-induced cardiomyocyte death and cardiac dysfunction. Here, we tested this hypothesis by examining the role of TCTP in cardiomyocyte-specific TCTP-overexpressing mice, and by investigating the effect of dihydroartemisinin (DHA), a pharmacological TCTP inhibitor^16,34,35^. Our results indicate a pivotal role of TCTP in cardiomyocyte survival, and also suggest that TCTP can prevent DOX-induced cardiac dysfunction.

## Materials and methods

An expanded Materials and methods section is available in the Supplementary Materials and Methods.

### Cell culture

Primary cultures of neonatal rat ventricular myocytes (NRVMs) and neonatal rat cardiac fibroblasts (NRCFs) were prepared from the heart of 3-day-old Wistar rats as previously described^4,36^. H9C2 cells were seeded and cultured in Dulbecco’s modified Eagle’s medium (DMEM) containing 10% fetal bovine serum (FBS) and a 1% solution of penicillin-streptomycin at 37 °C in 5% CO_2_. The next day, the medium was replaced with serum-free medium.

### Immunofluorescence microscopy

Primary NRVMs were seeded on coverslips. Mito Tracker Red (Thermo Fisher) was added to the medium 45 min before fixation. Cells were then fixed in 4% paraformaldehyde at room temperature for 15 min, permeabilized with 0.2% Triton X-100 for 5 min, and blocked with 5% BSA in PBS for 1 h. Cells were incubated with primary antibodies at 4 °C overnight, followed by 1 h incubation with secondary antibody (goat anti-rabbit AlexaFluor 488) and 5 min incubation with DAPI. Images were analyzed by deconvolution microscopy (Nikon).

### Cell death assay

The staining solution contained 2 μM Calcein-AM (Dojindo) and 2 μM ethidium homodimer-1 (Takara Bio) in serum-free medium. Cells were gently washed twice and then incubated with the staining solution under 5% CO_2_ in humidified air at 37 °C for 45 min. The live cells were stained with Calcein-AM (green) and dead cells with ethidium homodimer-1 (red). Cell death (%) was calculated from the numbers of live and dead cells^29,32^.

### Flow cytometry

Cells were washed twice with cold PBS and resuspended in Binding Buffer (BD). After incubation with APC Annexin V (BD) and 7-amino-actinomycin D (7-AAD) (BD) for 15 min at room temperature in the dark, apoptotic cells were quantified by flow cytometry. Annexin V-stained cells were considered to be apoptotic^37^.

### Mitochondrial permeability transition pore (mPTP) opening

Neonatal rat ventricular myocytes were incubated for 20 min with acetoxymethyl ester of Calcein-AM (1 μM) and then washed in the presence of CoCl_2_ (1 mM) for a further 20 min to remove the dye from the cytosolic compartment^30,32^. The loss of Calcein-AM fluorescence was used as an indicator of mPTP opening.

### Detection of Ad-LC3-GFP

NRVMs were transduced with adenovirus harboring LC3-GFP (Ad-LC3-GFP) as previously described^38,39^. The fluorescence of GFP-LC3 was observed under a fluorescence microscope. The number of GFP dots was determined by counting fluorescent puncta from at least three independent myocyte preparations. At least 60 cells were scored for each group.

### Mice

We generated TCTP transgenic mice (TCTP TG) with cardiac-specific overexpression of TCTP using α-myosin heavy chain (α-MHC) promoter on a C57BL/6 background (TCTP TG; Accession No. CDB0532T: http://www2.clst.riken.jp/arg/TG%20mutant%20mice%20list.html)^40^.

p53 knockout mice (p53 KO) (on a C57BL/6 background) were purchased from RIKEN BRC.

All animal experiments were conducted in accordance with the guidelines of the animal experiment committee of Yokohama City University and of Institutional Animal Care and Use Committee (IACUC) of RIKEN Kobe Branch.

### Mouse models

#### DOX-induced heart failure

Two- to three-month-old male mice were given intraperitoneal injection of 3 mg/kg DOX three times a week up to a total dose of 24 mg/kg^41,42^. At 5 weeks after the first injection, cardiac morphology and function were evaluated by echocardiography and catheterization.

#### ISO-induced heart failure model

Two- to three-month-old male C57BL/6 mice were given chronic ISO (Sigma–Aldrich) infusion via an osmotic mini-pump (DURECT Corporation) at a dose of 60 mg/kg/day for 2 or 7 days^4^.

#### Transverse aortic constriction (TAC)-induced heart failure model

Two- to three-month-old male C57BL/6 mice were anesthetized with isoflurane vapor titrated to maintain the lightest anesthesia possible. On average, 1.5% vol/vol isoflurane vapor was required to maintain adequate anesthesia. The animals were ventilated via tracheal intubation with a tidal volume of 0.5 ml and a respiratory rate of 90 breaths per minute. The left side of the chest was opened at the second intercostal space, and TAC or sham operation was performed^4^.

### Echocardiography

Mice were anesthetized with inhaled isoflurane (1.5%) using an induction chamber. Echocardiography was performed as previously described^4,9^.

### Cardiac catheterization

Mice were anesthetized with inhaled isoflurane (1.5%) and a 1.4F catheter (Millar) was inserted into the LV through the carotid artery. Hemodynamic measurements were performed as previously described^4^.

### Histological analysis

Heart specimens were fixed with formalin, embedded in paraffin, and sectioned at 3.5 μm. Sections were deparaffinized and fibrosis was evaluated by Masson-trichrome staining using the Accustatin Trichrome Stain Kit (Sigma–Aldrich)^4,5^. Apoptosis was determined by TUNEL staining using the DeadEnd fluorometric TUNEL system (Promega). Nuclei were stained with DAPI^4,5^. The numbers of TUNEL-positive nuclei and total nuclei were counted.

### Statistics

All data were expressed as mean ± standard error of the mean (S.E.M.). Comparison of data was performed using Student’s *t*-test for two groups. Multiple comparisons were made using one-way analysis of variance (ANOVA) followed by Tukey’s test or two-way ANOVA followed by Bonferroni’s post hoc test. For all analytical studies, the criterion of significance was assigned as *P* < 0.05.

## Results

### TCTP expression in cardiomyocytes

**Fig. 1:**
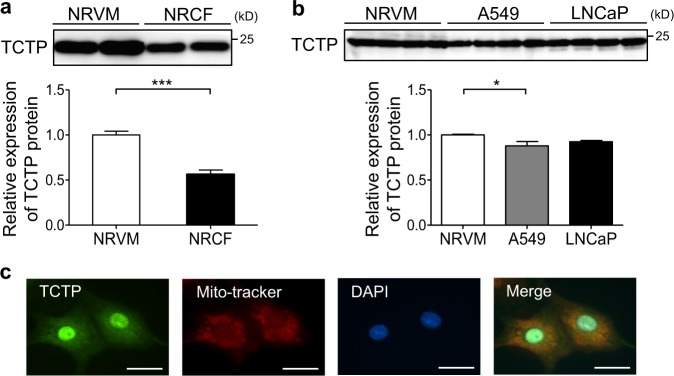
TCTP expression in cardiomyocytes. **a** TCTP protein expression in NRVMs and NRCFs. Ten microgram of total protein was loaded per lane (*n* = 4). **b** TCTP protein expression in NRVMs, A549, and LNCaP cells. Ten microgram of total protein was loaded per lane (*n* = 4). **c** To visualize mitochondria in NRVMs, MitoTracker Red (red) was added to the medium 45 min before fixation. After immunostaining with anti-TCTP antibody (green), cells were counterstained with DAPI (blue). Scale bar, 20 μm. *P < 0.05, ***P < 0.001. Unpaired, two-tailed Student’s *t*-test (**a**) or One-way ANOVA followed by Tukey’s test (**b**)

We examined the TCTP protein expression level in cardiomyocytes and cardiac fibroblasts. The TCTP expression level per total protein was 1.8-fold greater in NRVMs than in NRCFs (Fig. 1a). In addition, the expression level in NRVMs was similar to those in the cancer cell lines for which a pivotal role of cardiomyocyte in cell survival has been reported^22,24^ (Fig. 1b). TCTP was widely localized in nucleus, mitochondria, and cytoplasm of cardiomyocytes (Fig. 1c).

### TCTP downregulation resulted in cardiomyocyte death

**Fig. 2:**
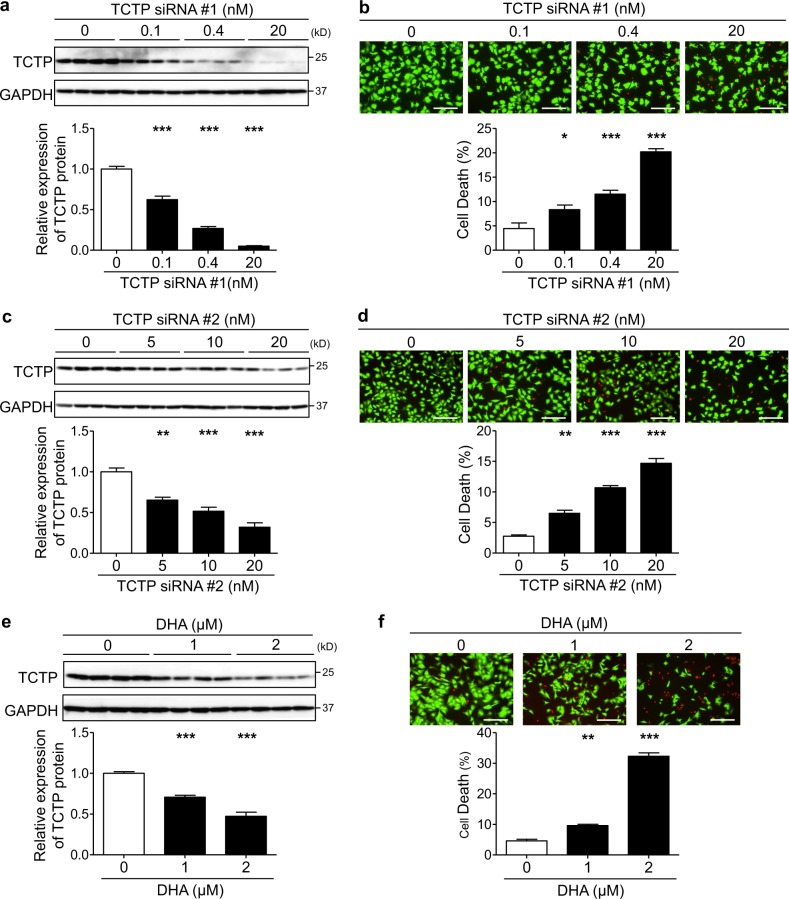
TCTP downregulation resulted in cardiomyocyte death. **a**–**d** NRVMs were transfected with various doses of TCTP siRNA #1 or siRNA #2 for 72 h. **e**, **f** NRVMs were treated with various concentrations of DHA for 48 h. **a**, **c**, **e** Protein expression of TCTP and GAPDH was analyzed after TCTP siRNAs transfection or DHA treatment (*n* = 4). **b**, **d**, **f** Cell death was determined by calcein-AM/ethidium homodimer-1 staining (*n* = 4). Live and dead cells were distinguished by calcein-AM (green) and ethidium homodimer-1 (red) staining, respectively. Scale bar, 200 μm. *P < 0.05, **P < 0.01, ***P < 0.001 vs 0. One-way ANOVA followed by Tukey’s test

In order to investigate the role of TCTP in cardiomyocyte survival, we downregulated TCTP expression with two different siRNAs (TCTP siRNA #1 and #2) in NRVMs. The dose-dependent effect of these TCTP siRNAs was confirmed by western blotting (Fig. 2a, c). Both TCTP siRNA #1 and #2 induced cardiomyocyte death in a dose-dependent manner (Fig. 2b, d). Furthermore, DHA, a pharmacological TCTP inhibitor, induced cardiomyocyte death accompanied by TCTP downregulation (Fig. 2e, f). Interestingly, however, TCTP siRNA did not reduce the viability of NRCFs (Fig. S1), suggesting that the role of TCTP in cell survival is cell-type-dependent.

### TCTP downregulation caused cardiomyocyte death through a Bnip3-dependent mechanism

**Fig. 3:**
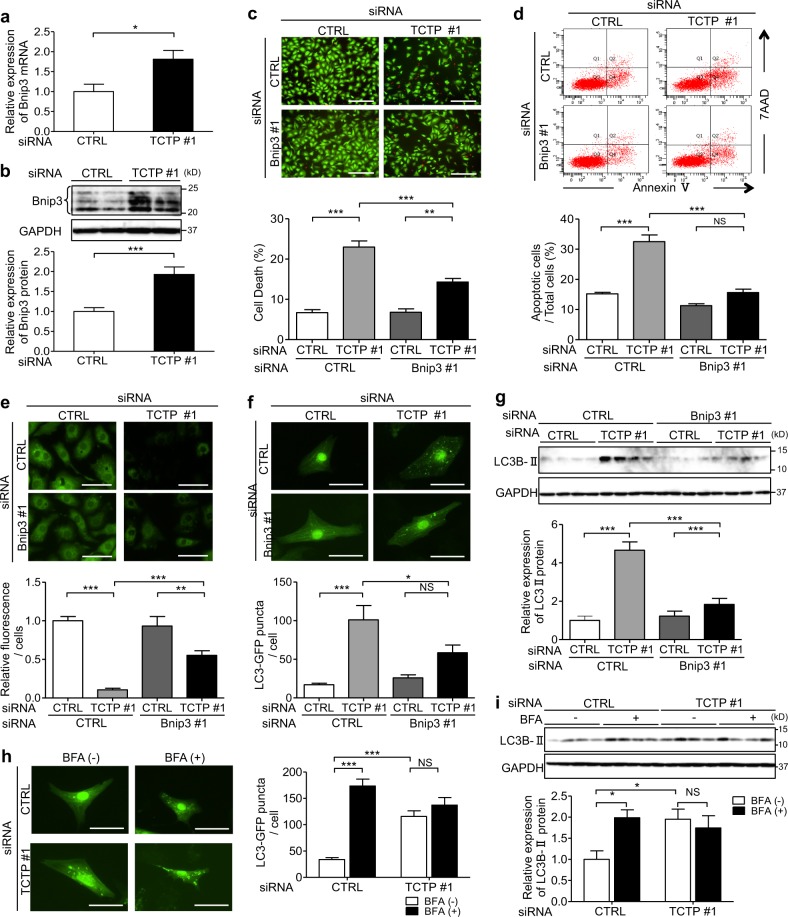
TCTP downregulation caused cardiomyocyte death through a Bnip3-dependent mechanism. **a**, **b** mRNA level (**a**) (*n* = 5–6) and protein expression (**b**) (*n* = 8) of Bnip3 in NRVMs transfected with non-targeting siRNA (CTRL siRNA) or siRNA targeting TCTP (TCTP siRNA #1) for 72 h. **c**–**e** NRVMs were transfected with non-targeting siRNA (CTRL siRNA) or siRNA targeting TCTP (TCTP siRNA #1), or Bnip3 (Bnip3 siRNA #1), or a mixture of both (TCTP siRNA #1 & Bnip3 siRNA #1). Cell death (**c**) (*n* = 4) and apoptosis (**d**) (*n* = 4–5) were determined by calcein-AM (green)/ethidium homodimer-1 (red) staining and flow cytometry after siRNA transfection for 72 h. Scale bar, 200 μm. e mPTP opening was assessed by co-loading with calcein/AM and CoCl2 after siRNA transfection for 24 h. Loss of green fluorescence is indicative of mPTP opening (*n* = 6). Scale bar, 40 μm. **f** NRVMs were infected with Ad-LC3-GFP (30 MOI) for 8 h and then transfected with non-targeting siRNA or siRNA targeting TCTP (TCTP siRNA #1), or Bnip3 (Bnip3 siRNA #1) or a mixture of both (TCTP siRNA #1 & Bnip3 siRNA #1) for 48 h. Green puncta indicate autophagosomes (*n* = 6). Scale bar, 25 μm. **g** Expression of LC3BII and GAPDH proteins in NRVMs transfection with siRNA for 72 h (*n* = 4). **h** NRVMs were infected with Ad-LC3-GFP (30 MOI) for 8 h and then transfected with non-targeting siRNA (CTRL siRNA) or siRNA targeting TCTP (TCTP siRNA #1) with or without BFA treatment for 6 h. Autophagosome formation was evaluated after 48 h. Green puncta indicate autophagosomes (*n* = 4). Scale bar, 25 μm. **i** Expression of LC3BII and GAPDH proteins in NRVMs transfection with siRNA for 72 h (*n* = 4). *P < 0.05, **P < 0.01, ***P < 0.001. Unpaired, two-tailed Student’s *t*-test (**a**, **b**) or One-way ANOVA followed by Tukey’s test (**c**–**g**) or two-way ANOVA followed by Bonferroni’s test (**h**, **i**)

To investigate the mechanism of TCTP-loss-induced cardiomyocyte death, we examined the characteristics of cardiomyocyte death, including annexin V expression, DNA fragmentation, mPTP opening, autophagosome accumulation, and LC3B II expression, in NRVMs after siRNA silencing of TCTP (Fig. 3d–g; Figs. S2b, S3d–f). All of these features were induced by TCTP silencing, suggesting that the TCTP-loss-induced cardiomyocyte death shows apoptotic and autophagic features, accompanied with mPTP opening. Recent reports indicate that Bnip3-induced cell death shows the same features^27–29^. Here, we found that TCTP downregulation increased Bnip3 expression at both the mRNA (1.8-fold) and protein levels (1.9-fold) in NRVMs (Fig. 3a, b; Fig. S3a).

Therefore, to examine the role of Bnip3 induction in the TCTP-loss-induced cardiomyocyte death, we downregulated Bnip3 expression in NRVMs by treatment with siRNAs (Figs. S2a, S3b). Bnip3 silencing significantly attenuated TCTP-loss-induced cardiomyocyte death (TCTP siRNA vs. TCTP+Bnip3 siRNA: 22.8% vs. 14.3%), apoptosis (TCTP siRNA vs. TCTP+Bnip3 siRNA: 32.5% vs. 15.6%), mPTP opening (Relative fluorescence intensity/cell: TCTP siRNA vs. TCTP+Bnip3 siRNA: 0.1 vs. 0.5), and autophagosome accumulation (LC3-GFP puncta/cell: TCTP siRNA vs. TCTP+Bnip3 siRNA: 101.0 vs. 58.4; Relative expression levels of LC3B II normalized to GAPDH: TCTP siRNA vs. TCTP siRNA+Bnip3 siRNA: 4.7 vs. 1.8) (Fig. 3c–g; Fig. S2b). Similar results were obtained with another Bnip3 siRNA (Bnip3 siRNA #2) (Fig. S3c, d, f). These findings suggested that Bnip3 induction is a major step in the pathway of TCTP-loss-induced cardiomyocyte death.

In this experiment, we did not observe significant attenuation of TCTP siRNA #2-induced mPTP opening by Bnip3 siRNA #2 (Fig. S3e). TCTP siRNA #1 downregulated TCTP expression more effectively than TCTP siRNA #2. Consistently with this, induction of mPTP opening by TCTP siRNA #1 was greater than that by TCTP siRNA #2 (Fig. 3e; Fig. S3e, g). Thus, the difference of downregulating effect between these two TCTP siRNAs may account for the difference in the results. Bnip3 may play a role in TCTP-loss-induced mPTP opening when TCTP is highly suppressed.

Among several proteins that are reported to regulate Bnip3 expression^43–45^, we found NF-kB protein expression was 54% decreased by TCTP siRNA (Fig. S4a). On the other hand, no significant change was observed in the expression of p53, Bax, E2F1, or FOXO3a (Fig. S4b–f). These findings suggested that NF-kB may be involved in the TCTP-loss-induced increase of Bnip3 expression.

Overall, our results indicate that TCTP loss caused cardiomyocyte death at least in part through a Bnip3-dependent mechanism.

Since there is controversy regarding the role of autophagy in cardiomyocyte death^46,47^, we examined its role in the TCTP-loss-induced cardiomyocyte death. In our studies, inhibition of autophagy by 3-MA or Atg5 siRNA resulted in suppression of TCTP-loss-induced cardiomyocyte death (Fig. S5a, b), suggesting that the autophagy under these conditions was maladaptive for cell survival.

Next, we examined the mechanism of the TCTP-loss-induced autophagosome accumulation. Autophagosome accumulation can result from either enhancement of autophagosome formation or inhibition of degradation^48^. BFA, an inhibitor of vacuolar H^+^-ATPase, disrupts autophagy processing by inhibiting autophagosome-lysosome fusion. In the presence of BFA, TCTP silencing induced no increase in autophagosome number or LC3B II protein expression in cardiomyocytes (Fig. 3h, i). These findings indicate that TCTP-loss-induced autophagosome accumulation was caused by inhibition of autophagosomal degradation, as has been observed in DOX-treated cardiomyocytes^48^.

### DOX treatment suppressed cardiac TCTP expression both in vitro and in vivo

**Fig. 4:**
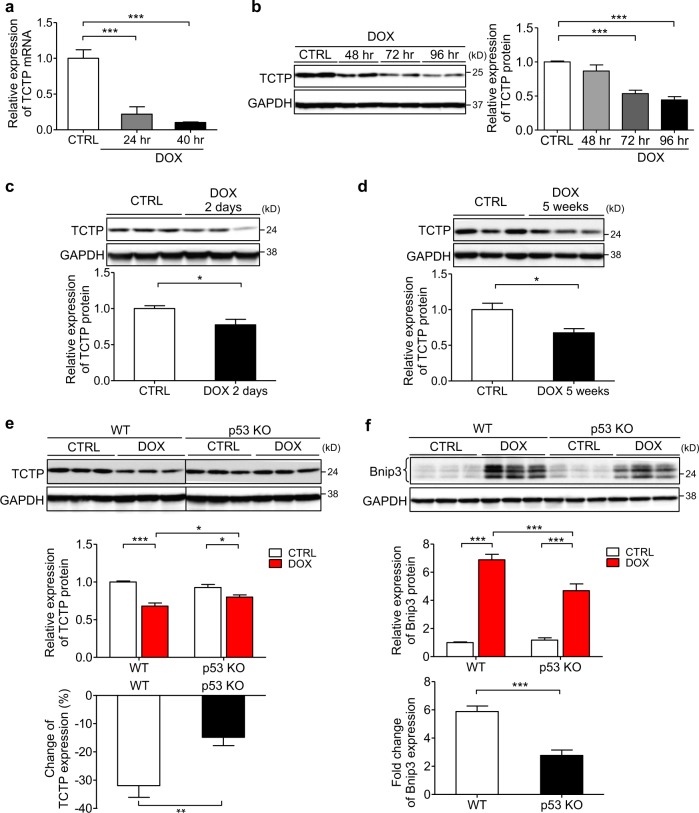
DOX treatment suppressed TCTP expression both in vivo and in vitro. **a**, **b** mRNA level (**a**) and protein expression (**b**) of TCTP in NRVMs treated with or without DOX (0.1 μM) for different times (*n* = 4). **c** C57BL/6 mice were treated intraperitoneally with DOX (20 mg/kg) or normal saline (CTRL). Protein expression of TCTP and GAPDH in hearts was analyzed after DOX treatment for 48 h (*n* = 7–8). **d** C57BL/6 mice were treated intraperitoneally with DOX (3 mg/kg) or normal saline. DOX was administered three times a week, with a total dose of 24 mg/kg. At 5 weeks after the first injection, protein expression of TCTP and GAPDH in hearts was analyzed (*n* = 4). **e**, **f** WT and p53 KO mice were treated intraperitoneally with DOX (20 mg/kg) or normal saline (CTRL). Protein expression of TCTP (**e**) (*n* = 11–12), Bnip3 (**f**) (*n* = 5~6) and GAPDH in hearts was analyzed after 48 h. *P < 0.05, **P < 0.01, ***P < 0.001. One-way ANOVA followed by Tukey’s test (**a**, **b**) or unpaired, two-tailed Student’s *t*-test (**c**, **d**, **e** <bottom> , f <bottom>) or two-way ANOVA followed by Bonferroni’s test (**e** <top> , f <top>)

To investigate the clinical significance of TCTP expression in the heart, we examined the TCTP expression level in cardiomyocytes and heart tissues after DOX treatment. We found that DOX treatment markedly suppressed the TCTP mRNA and protein expression in cultured NRVMs (Fig. 4a, b). In addition, TCTP expression was suppressed in the heart tissues after both acute (22.6%) and chronic (32.4%) DOX treatment (Fig. 4c, d). These findings indicated that TCTP downregulation may be involved in DOX-induced heart failure. On the other hand, conversely, TCTP was upregulated after chronic isoproterenol (ISO) infusion or chronic pressure overload induced by transverse aortic constriction (TAC) in mice (Fig. S6a–d). In accordance with this, TCTP mRNA and protein expression were increased by ISO treatment in cultured cardiomyocytes (Fig. S6e–g).

p53, a major tumor suppressor, is involved in the development of various physiological dysfunctions, including cardiac diseases such as heart failure^2,49^, ischemic heart disease^50^, and DOX-induced heart failure^41,51,52^. It was demonstrated that TCTP and p53 negatively regulate each other’s functions and expression levels^15,53,54^. To examine the role of p53 in DOX-induced TCTP downregulation, we examined cardiac TCTP expression in the heart of p53-deficient mice (p53 KO) after DOX treatment. The DOX-induced TCTP downregulation was attenuated in p53 KO mice (WT vs. p53KO: −31.9% vs. −14.8%) (Fig. 4e), indicating that DOX suppressed TCTP expression at least in part through a p53-dependent mechanism. In addition, we found that the DOX-induced increase of Bnip3 expression was attenuated in p53 KO mouse heart (WT vs. p53KO: 5.8-fold vs. 2.8-fold) (Fig. 4f). Overall, these findings suggest that p53 is involved in DOX-induced TCTP loss and enhancement of Bnip3 expression.

### Exogenous supplementation of TCTP rescued cardiomyocytes from DOX-induced death

**Fig. 5:**
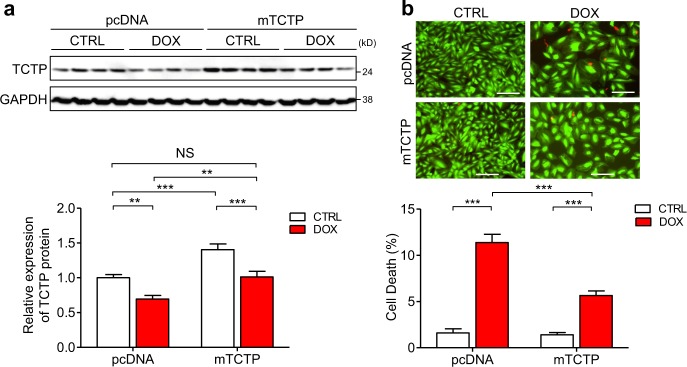
Exogenous supplementation of TCTP rescued DOX-induced cardiomyocyte death. H9C2 cells were transfected with pcDNA3.1-control vector or pcDNA3.1 mouse TCTP expression plasmid (mTCTP) for 24 h and then treated with DOX (0.1 μM) or vehicle for 48 h. **a** Protein expression of TCTP and GAPDH was analyzed by western blotting (*n* = 8). **b** Cell death was determined by calcein-AM/ethidium homodimer-1 staining (*n* = 4). Live and dead cells were distinguished by calcein-AM (green) and ethidium homodimer-1 (red) staining, respectively. Scale bar, 200 μm. *P < 0.05, **P < 0.01, ***P < 0.001. Two-way ANOVA followed by Bonferroni’s test

To investigate the significance of DOX-induced TCTP loss in relation to DOX’s cardiotoxicity, we examined the effect of exogenous supplementation of TCTP on DOX-induced cell death in a cardiomyocyte cell line, H9C2. DOX treatment induced TCTP downregulation (31% lower) and cell death (CTRL vs. DOX: 1.6% vs. 11.4%) (Fig. 5), while exogenous supplementation of TCTP via plasmid transfection significantly suppressed DOX-induced cell death (pcDNA vs. mTCTP: 11.4% vs. 5.7%) (Fig. 5a, b). Importantly, just supplementary exogenous expression, which rescues the DOX-induced TCTP loss, was enough to significantly inhibit DOX-induced cell death, suggesting that TCTP downregulation may play a key role in DOX-induced cardiomyocyte death.

### Cardiomyocyte-specific TCTP overexpression protected mice against DOX-induced cardiac dysfunction

**Fig. 6:**
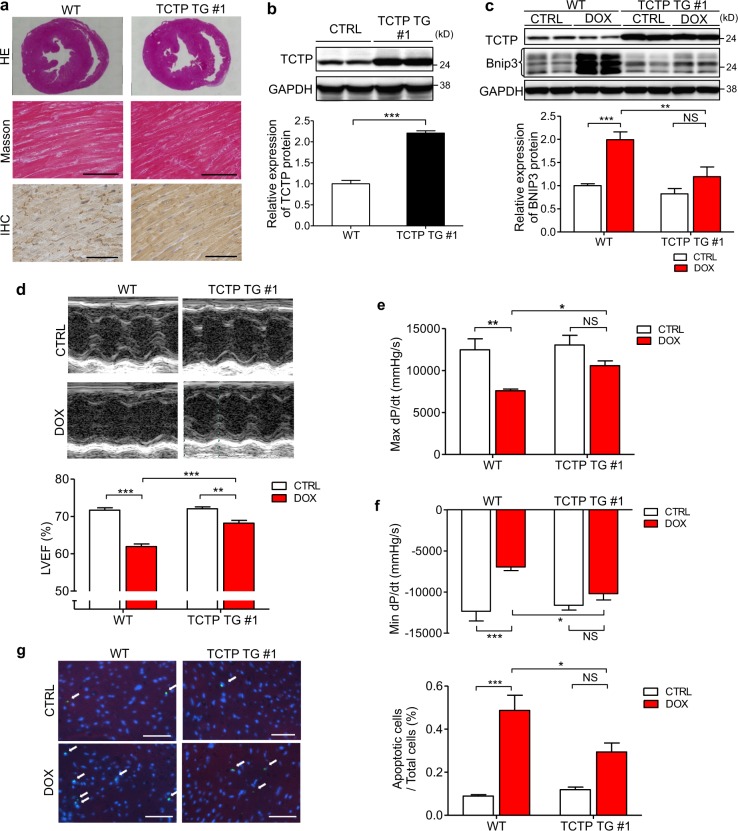
Cardiomyocyte-specific TCTP overexpression protected mice against DOX-induced cardiac dysfunction. **a** Representative images of sections from WT and TCTP TG mouse hearts stained with hematoxylin and eosin (HE) (top) and Masson trichrome (middle). Immunohistochemistry (IHC) with TCTP antibody in heart tissues of WT and TCTP TG mice (bottom). Scale bar, 50 μm. **b** TCTP and GAPDH protein expression in the left ventricle of WT and TCTP TG #1 mice (*n* = 5). **c** WT and TCTP TG mice were treated intraperitoneally with DOX (3 mg/kg) or normal saline (CTRL). DOX was administered three times a week, with a total dose of 24 mg/kg. Protein expression levels of TCTP, Bnip3, and GAPDH in the hearts were analyzed 24 h after the treatment (*n* = 4–5). **d**–**g** five weeks after the first injection, LVEF was evaluated by echocardiography (**d**) (*n* = 6–9). Max dP/dt (**e**) and Min dP/dt (**f**) were evaluated by cardiac catheterization (*n* = 6–7). **g** Cardiac apoptosis in the myocardium was assessed by TUNEL assay in heart sections from WT and TCTP TG mice with DOX or normal saline (CTRL) treatment. Nuclei were stained with DAPI (blue). Representative images are shown to the left. The white arrows indicate apoptotic cells in the sections (*n* = 4–7). Scale bar, 50 μm. *P < 0.05, **P < 0.01, ***P < 0.001. Unpaired, two-tailed Student’s *t*-test (**b**) or two-way ANOVA followed by Bonferroni’s test (**c**–**g**)

To examine the role of TCTP in cardiomyocytes in vivo, we generated TCTP transgenic mice (TCTP TG #1 and #2) with cardiac-specific overexpression of TCTP using α-MHC promoter. TCTP expression in the heart was significant greater in TCTP TG #1 mice than in WT mice (Fig. 6b; Fig. S7a–h).

There were no significant differences between WT mice and TCTP TG #1 mice in cardiac morphology or functions at baseline (Fig. 6a, d–f; Table S2).

We examined the effect of TCTP overexpression in cardiomyocytes on the DOX-induced cardiac dysfunction in mice. DOX-induced cardiac dysfunction was significantly attenuated in TCTP TG #1 mice (Fig. 6d–f; Table S2). Indexes of cardiac function, including LVEF and maximum and minimum dP/dt, were significantly better in DOX-treated TCTP TG mice than in WT mice (LVEF: WT vs. TCTP TG: 61.9% vs. 68.2%; Max dP/dt: WT vs. TCTP TG: 7592 mmHg/s vs.10581 mmHg/s; Min dP/dt: WT vs. TCTP TG: −6962 mmHg/s vs. −10191 mmHg/s). These findings indicate that TCTP overexpression prevented DOX-induced cardiac dysfunction. The effect of TCTP overexpression on DOX-induced cardiac dysfunction was confirmed in TCTP TG #2 mice, another mouse line of TCTP TG with milder overexpression of TCTP (Fig. S8a–d; Table S3). DOX treatment caused a significant increase of apoptotic cell death in the heart (Fig. 6g). Importantly, in accordance with the results of functional analysis, apoptotic cell death was significantly attenuated in hearts from TCTP TG #1 compared with WT (WT vs. TCTP TG: 0.49% vs. 0.29%). In addition, we found that Bnip3 expression was increased, accompanied with downregulation of TCTP, in the hearts of both WT and TCTP TG #1 mice. However, the increase of Bnip3 expression was significantly smaller in hearts from TCTP TG #1 mice (WT vs. TCTP TG: 2.0-fold vs. 1.2-fold) (Fig. 6c), suggesting that TCTP loss may be related to DOX-induced Bnip3 expression.

### Treatment with DHA, a pharmacological inhibitor of TCTP, resulted in cardiac dysfunction in mice

**Fig. 7:**
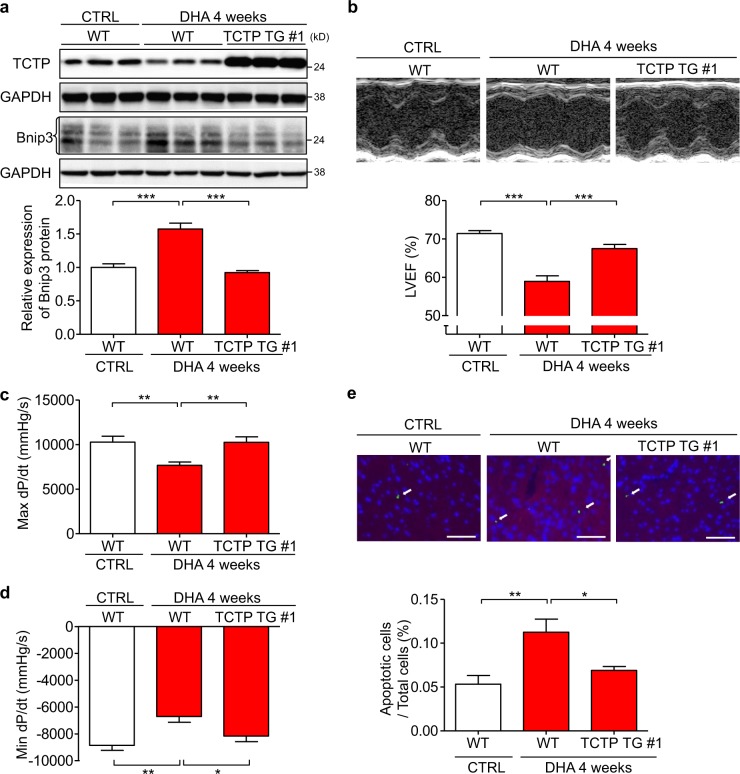
DHA treatment resulted in cardiac dysfunction accompanied with reduced expression of TCTP in the heart. WT and TCTP TG mice were treated intraperitoneally with DHA (30 mg/kg) or normal saline (CTRL) once a day for 4 weeks. **a** Protein expression levels of TCTP, Bnip3, and GAPDH in the heart were analyzed by western blotting (*n* = 5–7). **b** LVEF was evaluated by echocardiography (*n* = 10–11). **c**, **d** Max dP/dt and Min dP/dt were evaluated by cardiac catheterization (*n* = 9–11). **e** Cardiac apoptosis in the myocardium was assessed by TUNEL assay in heart sections from WT and TCTP TG mice given DHA or vehicle treatment. Nuclei were stained with DAPI (blue). Representative images are shown to the left. The white arrows indicate apoptotic cells in the sections (*n* = 5). Scale bar, 50 μm. *P < 0.05, **P < 0.01, ***P < 0.001. One-way ANOVA followed by Tukey’s test

To investigate the effect of TCTP loss on cardiac function in vivo, we treated mice with DHA, a pharmacological inhibitor of TCTP (Fig. 2e). DHA suppressed TCTP and enhanced Bnip3 protein expression in mouse heart. In addition, the extent of Bnip3 induction by DHA was less in TCTP TG #1 than in WT mice (Fig. 7a), indicating that DHA-induced TCTP loss may be involved in the DHA-induced increase of Bnip3 expression. Importantly, we found that DHA treatment caused heart failure. In addition, cardiac overexpression of TCTP significantly attenuated the DHA-induced left ventricular dysfunction (LVEF: WT vs. TCTP TG: 58.9% vs. 67.5%; Max dP/dt: WT vs. TCTP TG: 7670 mmHg/s vs.10262 mmHg/s; Min dP/dt: WT vs. TCTP TG: −6705 mmHg/s vs.−8162 mmHg/s) (Fig. 7b–d; Table S4). DHA also caused a significant increase of apoptotic cell death in the heart, and this was significantly attenuated by TCTP overexpression in cardiomyocytes (WT vs. TCTP TG: 0.11% vs. 0.07%) (Fig. 7e). These findings indicate that loss of TCTP may cause cardiomyocyte death and cardiac dysfunction in vivo.

## Discussion

To our knowledge, this is the first report on the function of TCTP in the heart, although TCTP is known to play important roles in cell survival in a cell-type-dependent manner^12,15,19,20^. Our results here support a pivotal role of TCTP in the maintenance of cardiomyocyte viability (Fig. S9). We also identified Bnip3 as a new player in TCTP-loss-induced cell death. TCTP downregulation by siRNA resulted in cardiomyocyte death, at least in part through a Bnip3-dependent mechanism (Fig. 3; Fig. S2 and S3). Bnip3 is one of the pro-apoptotic members of the BH-3-only subfamily of Bcl-2 family proteins. Although there are several reports on the relationship between TCTP and Bcl-2 family proteins, such as Bax, Bcl-xL, MCL-1, etc.^14^, this is the first report of the involvement of Bnip3 in TCTP-induced signaling. In accordance with this finding, TCTP-loss-induced cardiomyocyte death showed apoptotic and autophagic features accompanied with mPTP opening, resembling those reported in Bnip3-induced cell death^27,55^.

To examine the mechanisms underlying TCTP-loss-induced cardiomyocyte death, we first investigated p53-induced signaling. It is reported that TCTP and p53 mutually downregulate each other’s expression in cancer cells^53^, and p53 has been reported to play an important role in cardiac cell death^2^. However, a positive relationship between the expression levels of TCTP and p53 was reported in non-cancer tissues^56^. After TCTP siRNA treatment in NRVMs, we did not observe significant induction of p53 (Fig. S4b). In addition, protein expression of Bax (Fig. S4c), a pro-apoptotic protein, which is transactivated by p53, was rather suppressed by TCTP siRNA treatment. Notably, on the other hand, we observed the induction of Bnip3 in response to loss of TCTP (Fig. 3a, b; Fig. S3a). In addition, NF-kB, a negative regulator of Bnip3^43^, was downregulated by TCTP siRNA treatment, suggesting that attenuation of the inhibitory effect of NF-kB on Bnip3 expression may be involved in the TCTP-loss-induced Bnip3 expression. At least in this experimental system, Bnip3 plays a more important role than p53 in TCTP-loss-induced cell death.

In exploring the clinical significance of the TCTP expression in the heart, we found that DOX treatment suppressed TCTP expression in cultured cardiomyocytes and mouse heart. In addition, exogenous supplementation of TCTP significantly inhibited DOX-induced cardiomyocyte death (Fig. 4). Importantly, just supplementary exogenous expression to rescue the DOX-induced reduction of TCTP expression was enough to significantly inhibit DOX-induced cardiomyocyte death. These findings indicate that the TCTP downregulation itself may contribute the cardiotoxicity of DOX.

In line with these findings, cardiomyocyte-specific overexpression of TCTP significantly inhibited DOX-induced Bnip3 expression and the development of cardiac dysfunction in mice. TCTP overexpression in cardiomyocytes caused no significant changes in cardiac function or morphology. However, after DOX treatment, development of cardiac dysfunction was significantly attenuated in cardiomyocyte-specific TCTP-overexpressing mice (Fig. 6; Fig. S8). These findings suggested that maintenance of TCTP levels in cardiomyocytes may be a novel therapeutic strategy to ameliorate or prevent DOX-induced heart failure.

To examine the mechanism of DOX-induced TCTP loss, we assessed the effect of DOX treatment on cardiac TCTP and Bnip3 expression in mice. p53 plays an important role in the development of DOX-induced heart failure^41,51,52^, and as mentioned above, TCTP and p53 mutually downregulate each other’s expression^53^. Our results support the idea that p53 is involved in the DOX-induced TCTP loss and enhancement of Bnip3 expression in mouse heart.

To investigate the effect of TCTP loss on cardiac function in vivo, we next examined the effect of DHA, a TCTP pharmacological inhibitor, on cardiac function^16,34,35^. As expected, DHA treatment downregulated TCTP in the heart. In addition, DHA induced heart failure accompanied with an increase of apoptosis in cardiomyocytes, which were rescued by TCTP overexpression (Fig. 7). These findings indicate that the maintenance of TCTP expression may be important for cardiomyocyte survival and preservation of cardiac function. DHA is an established agent for the treatment of malaria infection^57^ Recently, mouse and canine studies indicated that long-term DHA therapy has an anti-tumor effect^58,59^, which appears to be due mainly to its pro-apoptotic action. To our knowledge, this is the first report regarding the effect of DHA on cardiac function. It may be important to consider this cardiac side effect, especially in patients receiving high-dose, long-term treatment with DHA. These findings suggest that maintenance of TCTP expression in cardiomyocytes would be important for the prevention of cardiomyocyte death and heart failure.

Several molecules such as p53^53^, CREB^60^, and chromodomain helicase/ATPase DNA binding protein 1-like gene (CHD1L)^61^ are reported to be involved in the regulation of TCTP expression. During the treatment of heart failure, signaling pathways that affect the functions of these proteins should be borne in mind. cAMP signaling is reported to upregulate TCTP expression in cancer cells^60^. CREB-induced transactivation is involved here. The concentration of catecholamines in blood is increased in patients with heart failure^62^. In accordance with this, cardiac TCTP expression was elevated in several mouse heart failure models, including a chronic catecholamine infusion model and the TAC model (Fig. S6). This response may act protectively against the development of heart failure by preventing stress-induced cardiomyocyte death. On the other hand, we can speculate that treatment with β-adrenergic receptor blockers, one of the established therapies for heart failure, may lead to a reduction of TCTP expression through its inhibitory effect on CREB function^63^. Consideration of this possible adverse effect may be helpful in the development of new treatments to prevent cardiomyocyte death. Interestingly, miRNA-27b downregulates TCTP protein expression^64^, and the therapeutic utility of miRNA inhibition by modified antisense oligonucleotides has been reported^65,66^. Induction of TCTP expression in this way could be a candidate for heart failure therapy.

Our findings indicate that TCTP plays a pivotal role in cardiomyocyte survival, at least in part through a Bnip3-dependent mechanism. DOX-induced TCTP loss may be involved in the cardiotoxicity of DOX. TCTP may be a candidate therapeutic target to prevent DOX-induced heart failure.

## Supplementary information

Supplemental information
