# Supplementary material for: Translationally controlled tumor protein (TCTP) plays a pivotal role in cardiomyocyte survival through a Bnip3-dependent mechanism
Source: Cell Death Dis. 2019 Jul 18;10(8):549. doi: 10.1038/s41419-019-1787-7 (PMC6639386; doi:10.1038/s41419-019-1787-7)
Supplement: Supplementary file 1 — Supplemental information [file 41419_2019_1787_MOESM1_ESM.pdf]

Supplementary materials and methods

Reagents and antibodies

Anti-HRF (TCTP) antibody (PM107) was purchased from Medical and Biological Laboratories, Co., Ltd. Anti-Bnip3 antibody (#3769), anti-LC3B antibody (#2775), anti-NF-kB p65 antibody (#3034), anti-p53 antibody (#2524) and anti-Bax (#2772) were purchased from Cell Signaling Technology. Anti-GAPDH antibody (sc-32233), anti-E2F1 antibody (sc-193), anti-FOXO3a antibody (sc-11351), and 3-methyladenine (3-MA), an inhibitor of class III phosphoinositide 3-kinase, were purchased from Santa Cruz Biotechnology. Bafilomycin A1 (BFA), a specific inhibitor of vacuolar proton ATPases, was purchased from Cayman Chemical. Isoproterenol (ISO) and DOX were purchased from Sigma-Aldrich. DHA was purchased from Tokyo Chemical Industry.

Cell culture

Primary cultures of neonatal rat ventricular myocytes (NRVMs) and neonatal rat cardiac fibroblasts (NRCFs) were prepared from the heart of three-day-old Wistar rats as previously described [1, 2]. H9C2 cells were seeded and cultured in Dulbecco's modified Eagle's medium (DMEM) containing 10% fetal bovine serum (FBS) and a 1% solution of penicillin-streptomycin at 37°C in 5% CO_2. The next day, the medium was replaced with serum-free medium.

Immunofluorescence microscopy

Primary NRVMs were seeded on coverslips. Mito Tracker Red (Thermo Fisher) was added to the medium 45 min before fixation. Cells were then fixed in 4% paraformaldehyde at room temperature for 15 min, permeabilized with 0.2% Triton X-100 for 5 min, and blocked with 5% BSA in PBS for 1 hr. Cells were incubated with primary antibodies at 4 °C overnight, followed by 1 hr incubation with secondary antibody (goat anti-rabbit AlexaFluor 488) and 5 min incubation with DAPI. Images were analyzed by deconvolution microscopy (Nikon).

Transfection of small interfering RNA (siRNA) and plasmid

Two kinds of double-stranded siRNAs of TCTP and Bnip3 were purchased from siGENOME or QIAGEN. Double-stranded siRNAs of Atg5 was purchased from Invitrogen. The siRNA sequences are shown in Table S1. NRVMs were transfected with siRNA using Lipofectamine RNAiMAX Transfection Reagent according to the manufacturer's instructions (Invitrogen).

To overexpress TCTP, mouse TCTP plasmid was transfected into H9C2 cells using Amaxa's Nucleofector device according to the manufacturer's instructions (Lonza).

Cell death assay

The staining solution contained 2 μM Calcein-AM (Dojindo) and 2 μM ethidium homodimer-1 (Takara Bio) in serum-free medium. Cells were gently washed twice and then incubated with the staining solution under 5% CO_2 in humidified air at 37°C for 45 min. The live cells were stained with Calcein-AM (green) and dead cells with ethidium homodimer-1 (red). Cell death (%) was calculated from the numbers of live and dead cells [3, 4].

MTT assay

MTT assay was performed to evaluate cell viability. Neonatal rat cardiac fibroblasts (NRCFs) were seeded onto 96-well tissue culture plates (4.8×10^3 cells per well) in DMEM with 10% FBS and 1% solution of penicillin-streptomycin for 24 hr and starved in DMEM-low glucose for 24 hours. Then, 10 μl of MTT solution (5 mg/ml) was added to each well, and incubation was continued for 4 hr. The amount of metabolized MTT was determined with a microplate reader [5].

Flow Cytometry

Cells were washed twice with cold PBS and resuspended in Binding Buffer (BD). After incubation with APC Annexin V (BD) and 7-amino-actinomycin (7-AAD) (BD) for 15 min at room temperature in the dark, apoptotic cells were quantified by flow cytometry. Annexin V-stained cells were considered to be apoptotic [6].

Mitochondrial Permeability Transition Pore (mPTP) Opening

Neonatal rat ventricular myocytes were incubated for 20 min with acetoxymethyl ester of Calcein-AM (1 µM) and then washed in the presence of $CoCl_2$ (1 mM) for a further 20 min to remove the dye from the cytosolic compartment [4, 7]. The loss of Calcein-AM fluorescence was used as an indicator of mPTP opening.

Detection of Ad-LC3-GFP

NRVMs were transduced with adenovirus harboring LC3-GFP (Ad-LC3-GFP) as previously described [8, 9]. The fluorescence of GFP-LC3 was observed under a fluorescence microscope. The number of GFP dots was determined by counting fluorescent puncta from at least three independent myocyte preparations. At least 60 cells were scored for each group.

Real-time quantitative PCR

Total RNA was extracted using RNAiso Plus according to the manufacturer's protocol (Takara Bio). RNA concentration and quality were determined by spectrophotometry. To remove contaminating genomic DNA, samples were treated with DNase I (Promega). Total RNA was reverse-transcribed with a PrimeScript™ RT reagent kit according to the manufacturer's instructions (Takara Bio). mRNA expression was quantified by quantitative real-time PCR using MyiQ2 (Bio-Rad) with SYBR Green. The amount of mRNA was normalized to that of 18S rRNA to obtain the relative amount. Primer sequences were as follows: rat Bnip3 (Eurofins): forward: 5'-GCCATTGGATTGGGGATCTAC, forward: 5'-ACTGTGTGAGCAGAAGGCAG [10], rat TCTP (Eurofins): forward: 5'-AAACCAGAAAGGGTAAAGCC-3', reverse: 5'-TCCACT CCAAATAAATCACGG-3' [11].

Mice

We generated TCTP transgenic mice (TCTP TG) with cardiac-specific overexpression of TCTP using α-myosin heavy chain (α-MHC) promoter on a C57BL/6 background (TCTP TG; Accession No. CDB0532T: http://www2.clst.riken.jp/arg/TG%20 mutant%20mice%20list.html) [12]. Mice were genotyped by PCR (forward primer, GTGGTGTAGGAAAGTCAGGA; reverse, GGTAGATGATCATGGTGGCA). The PCR conditions consisted of 94℃ for 1 minute, 33 cycles of 94℃ for 15 seconds each, 58℃ for 15 seconds, and 72℃ for 30 seconds, followed by 72℃ for 5 minutes.

p53 knockout mice (p53 KO) (on a C57BL/6 background) were purchased from RIKEN BRC. Mice were genotyped by PCR (forward primer: GTTATGCATCCATACAGTACA; reverse: CAGGATATCTTCTGGAGGAAG). The PCR conditions consisted of 94℃ for 5 minute, 34 cycles of 94℃ for 30 seconds each, 57℃ for 30 seconds, and 72℃ for 120 seconds, followed by 72℃ for 5 minutes.

All animal experiments were conducted in accordance with the guidelines of the animal experiment committee of Yokohama City University and of Institutional Animal Care and Use Committee (IACUC) of RIKEN Kobe Branch.

Western blotting

Tissues from animals were homogenized in lysis buffer (25 mM Tris-HCl (pH 7.5), 150 mM NaCl, 5 mM $MgCl_2$, 1 % NP-40, 5 % glycerol containing protease inhibitor (10 mM $Na_4P_2O_7$, 100 mM NaF, 10 mM Na_3VO_4, 20 μg/ml TLCK, 10 μg/ml leupeptin, 1 mM PMSF, 50 U ETI, 2 μg/ml aprotinin)) and centrifuged at 15, 000 rpm, for 15 minutes at 4℃. Samples were separated on 10 % SDS-polyacrylamide gels and transferred onto nitrocellulose membranes. Primary antibodies were applied overnight at 4℃. Immunoreactive protein bands were visualized with a Clarity Western ECL Substrate kit (Bio-Rad) or a Western Lightning ECL Pro kit (Perkin Elmer). Membranes were imaged with an image analyzer (Fujifilm).

Statistics

All data were expressed as mean ± standard error of the mean (S.E.M.). Comparison of data was performed using Student’s t test for 2 groups. Multiple comparisons were made using one-way analysis of variance (ANOVA) followed by Tukey's test or two-way ANOVA followed by Bonferroni’s post hoc test. For all analytical studies, the criterion of significance was assigned as $P<0.05$.

References

1. Okumura S, Fujita T, Cai W, Jin M, Namekata I, Mototani Y*, et al.* Epac1-dependent phospholamban phosphorylation mediates the cardiac response to stresses. *The Journal of clinical investigation* 2014, **124**(6)**:** 2785-2801.

2. Jin H, Fujita T, Jin M, Kurotani R, Hidaka Y, Cai W*, et al.* Epac activation inhibits IL-6-induced cardiac myocyte dysfunction. *The journal of physiological sciences : JPS* 2018, **68**(1)**:** 77-87.

3. Zhuo XZ, Wu Y, Ni YJ, Liu JH, Gong M, Wang XH*, et al.* Isoproterenol instigates cardiomyocyte apoptosis and heart failure via AMPK inactivation-mediated endoplasmic reticulum stress. *Apoptosis : an international journal on programmed cell death* 2013.

4. Dhingra R, Margulets V, Chowdhury SR, Thliveris J, Jassal D, Fernyhough P*, et al.* Bnip3 mediates doxorubicin-induced cardiac myocyte necrosis and mortality through changes in mitochondrial signaling. *Proc Natl Acad Sci U S A* 2014, **111**(51)**:** E5537-5544.

5. Chen Y, Fujita T, Zhang D, Doan H, Pinkaew D, Liu Z*, et al.* Physical and functional antagonism between tumor suppressor protein p53 and fortilin, an anti-apoptotic protein. *J Biol Chem* 2011, **286**(37)**:** 32575-32585.

6. Jia S, Qiao X, Ye J, Fang X, Xu C, Cao Y*, et al.* Nogo-C regulates cardiomyocyte apoptosis during mouse myocardial infarction. *Cell death & disease* 2016, **7**(10)**:** e2432.

7. Quinsay MN, Lee Y, Rikka S, Sayen MR, Molkentin JD, Gottlieb RA*, et al.* Bnip3 mediates permeabilization of mitochondria and release of cytochrome c via a novel mechanism. *J Mol Cell Cardiol* 2010, **48**(6)**:** 1146-1156.

8. Maejima Y, Kyoi S, Zhai P, Liu T, Li H, Ivessa A*, et al.* Mst1 inhibits autophagy by promoting the interaction between Beclin1 and Bcl-2. *Nat Med* 2013, **19**(11)**:** 1478-1488.

9. Ikeda Y, Shirakabe A, Maejima Y, Zhai P, Sciarretta S, Toli J*, et al.* Endogenous Drp1 mediates mitochondrial autophagy and protects the heart against energy stress. *Circulation research* 2015, **116**(2): 264-278.

10. Gallo S, Gatti S, Sala V, Albano R, Costelli P, Casanova E*, et al.* Agonist antibodies activating the Met receptor protect cardiomyoblasts from cobalt chloride-induced apoptosis and autophagy. *Cell Death Dis* 2014, **5:** e1185.

11. Zhu WL, Cheng HX, Han N, Liu DL, Zhu WX, Fan BL*, et al.* Messenger RNA expression of translationally controlled tumor protein (TCTP) in liver regeneration and cancer. *Anticancer Res* 2008, **28**(3A): 1575-1580.

12. Subramaniam A, Jones WK, Gulick J, Wert S, Neumann J, Robbins J. Tissue-specific regulation of the alpha-myosin heavy chain gene promoter in transgenic mice. *The Journal of biological chemistry* 1991, **266**(36): 24613-24620.

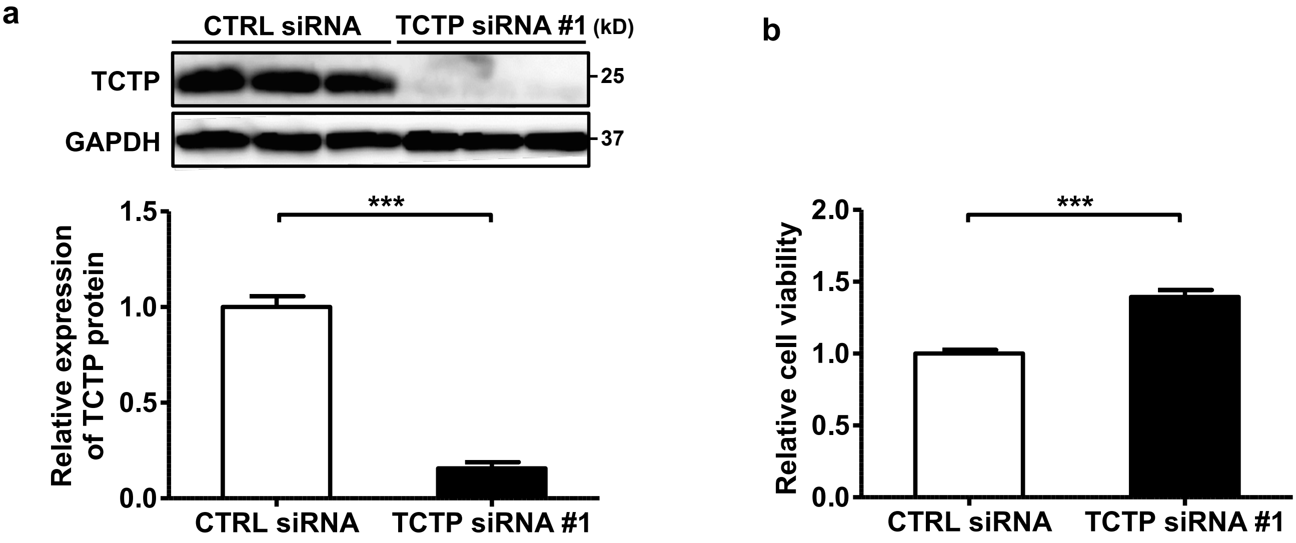

Fig. S1: TCTP down-regulation did not induce cardiac fibroblast death. NRCFs were transfected with non-targeting (CTRL) or TCTP-specific siRNA. Protein expression of TCTP **(a)** and cell viability **(b)** were analyzed by western blotting and MTT assay, respectively, after 72 hr (n=6). ****P*<0.001. Unpaired, two-tailed Student's *t* test.

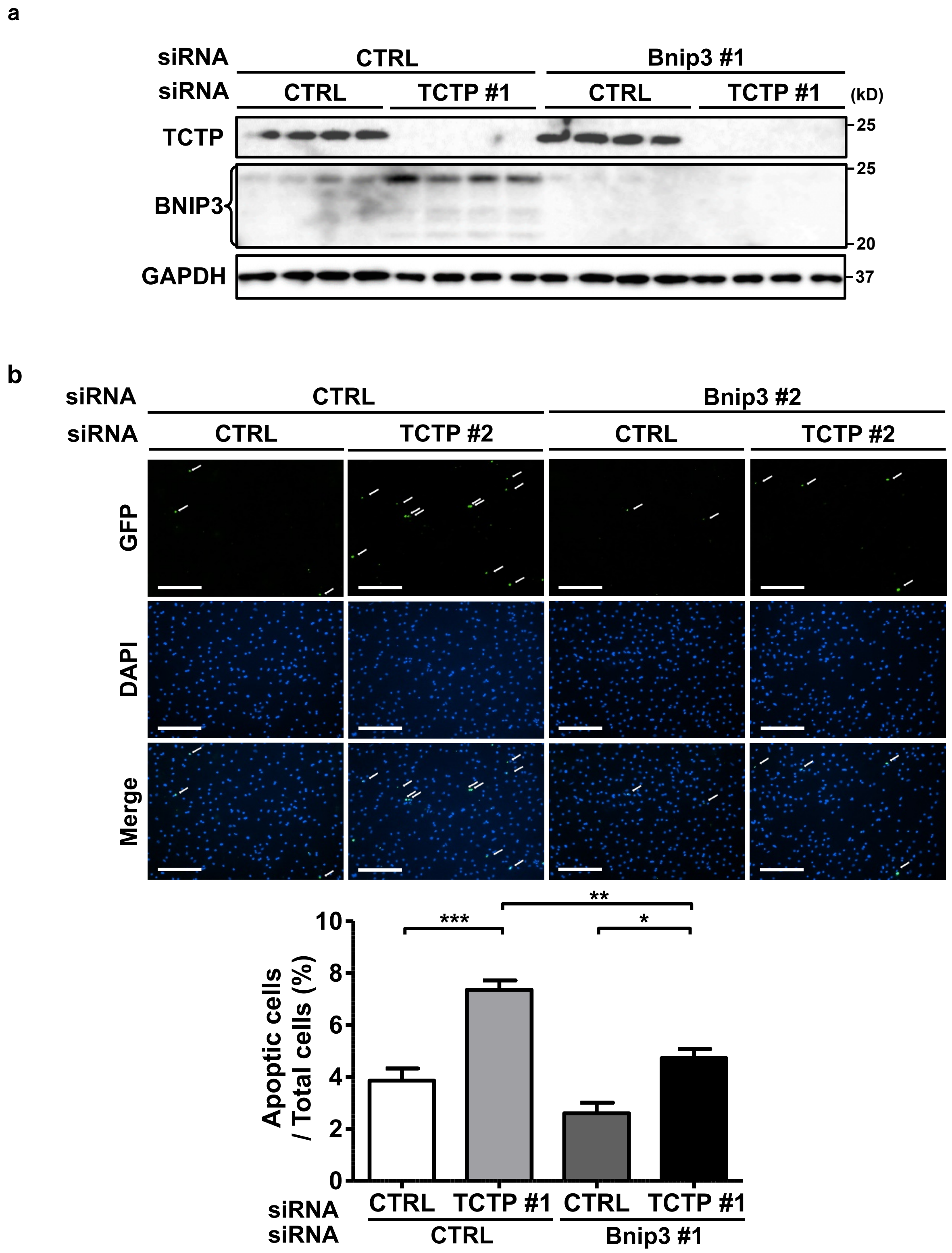

Fig. S2: TCTP down-regulation induced cardiomyocyte apoptosis through a BNIP3-dependent mechanism. NRVMs were transfected with non-targeting siRNA (CTRL siRNA) or siRNA targeting TCTP (TCTP siRNA #1), or BNIP3 (BNIP3 siRNA #1), or a mixture of both (TCTP siRNA #1 & BNIP3 siRNA #1). **a**, Expression of TCTP, Bnip3 and GAPDH proteins in NRVMs was analyzed after siRNA transfection for 72 hr. **b**, Apoptosis was determined by TUNEL assay after 72 hr. The white arrows indicate apoptotic cells (n=4). Scale bars, 200 μm. **P*<0.05, ***P*<0.01, ****P*<0.001. One-way ANOVA followed by Tukey's test **(b)**.

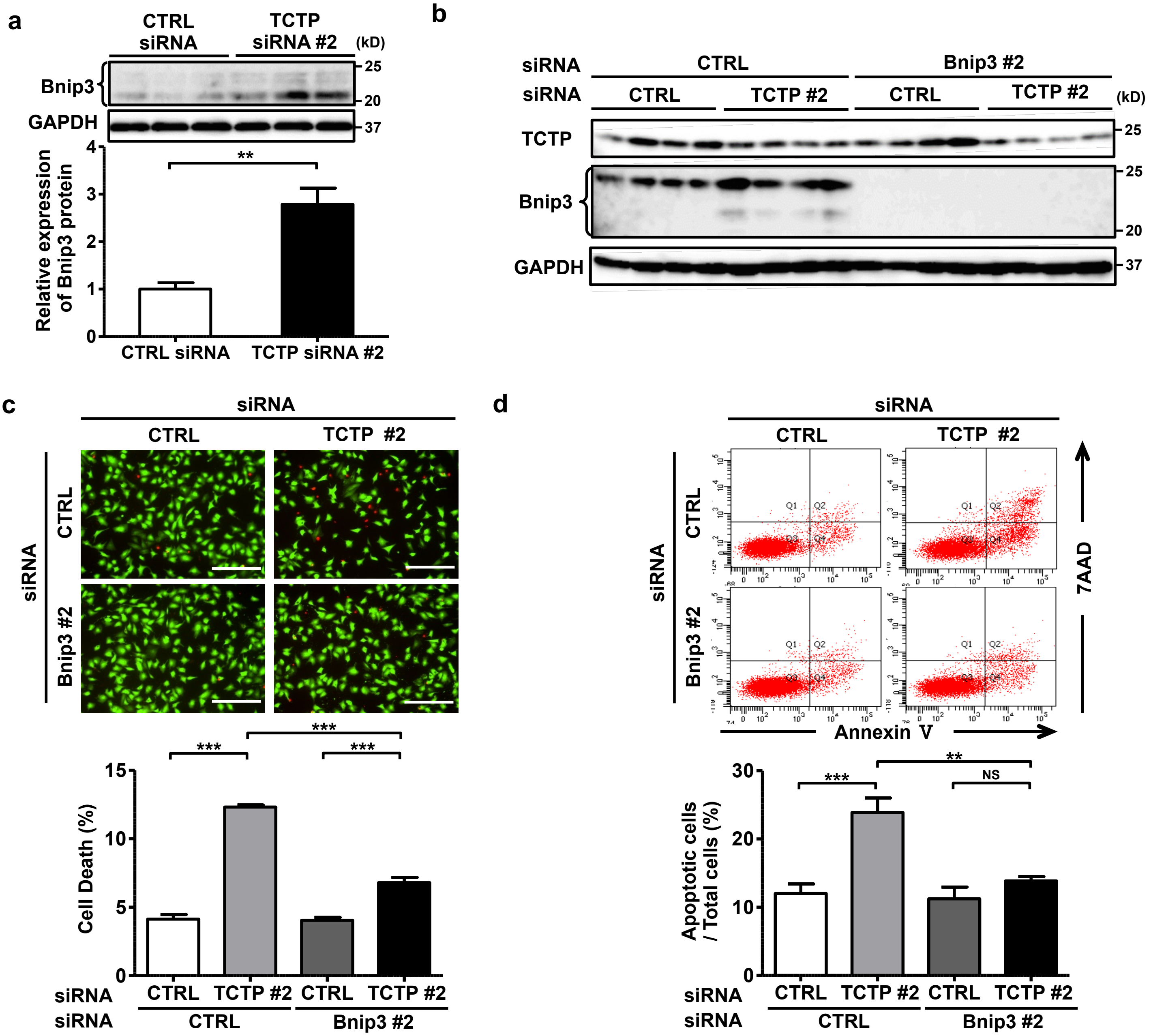
a
CTRL siRNA
TCTP siRNA #2
(kD)
Bnip3
GAPDH
25
20
37
Relative expression of Bnip3 protein
**
CTRL siRNA
TCTP siRNA #2
b
siRNA
CTRL
Bnip3 #2
siRNA
CTRL
TCTP #2
CTRL
TCTP #2
(kD)
TCTP
Bnip3
GAPDH
25
25
20
37
c
siRNA
CTRL
TCTP #2
siRNA
CTRL
Bnip3 #2
Cell Death (%)
***
***
***
siRNA
CTRL
TCTP #2
CTRL
TCTP #2
siRNA
CTRL
Bnip3 #2
d
siRNA
CTRL
TCTP #2
siRNA
CTRL
Bnip3 #2
Q1
Q2
Q3
Q4
7AAD
Annexin V
Apoptotic cells / Total cells (%)
***
**
NS
siRNA
CTRL
TCTP #2
CTRL
TCTP #2
siRNA
CTRL
Bnip3 #2

e

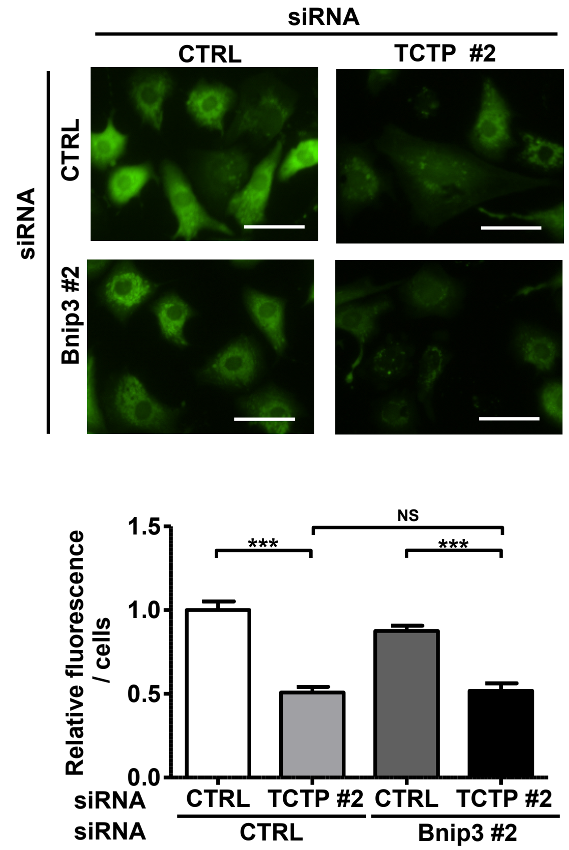
siRNA
CTRL
TCTP #2
siRNA
CTRL
Bnip3 #2
Relative fluorescence / cells
1.5
1.0
0.5
0.0
***
NS
***
siRNA CTRL TCTP #2 CTRL TCTP #2
siRNA CTRL Bnip3 #2

f

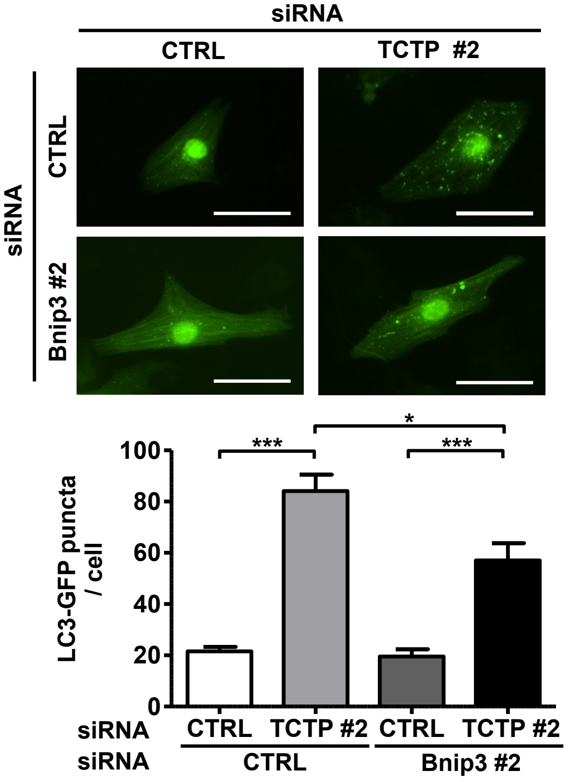
siRNA
CTRL
TCTP #2
siRNA
CTRL
Bnip3 #2
LC3-GFP puncta / cell
100
80
60
40
20
0
***
*
***
siRNA CTRL TCTP #2 CTRL TCTP #2
siRNA CTRL Bnip3 #2

g

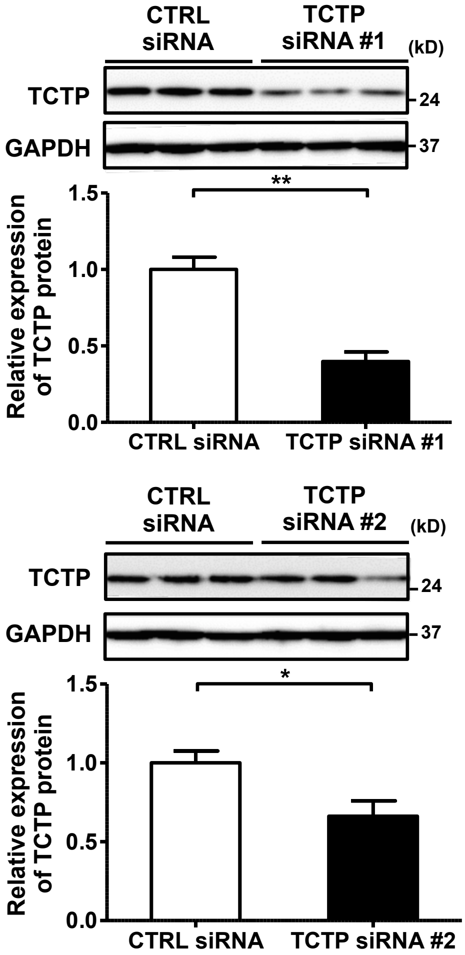
CTRL siRNA
TCTP siRNA #1
(kD)
TCTP
GAPDH
24
37
Relative expression of TCTP protein
1.5
1.0
0.5
0.0
**
CTRL siRNA
TCTP siRNA #1
CTRL siRNA
TCTP siRNA #2
(kD)
TCTP
GAPDH
24
37
Relative expression of TCTP protein
1.5
1.0
0.5
0.0
*
CTRL siRNA
TCTP siRNA #2

Fig. S3: TCTP down-regulation induced cardiomyocyte death through a BNIP3-dependent mechanism. a, Expression of Bnip3 protein in NRVMs transfected with non-targeting siRNA (CTRL siRNA) or siRNA targeting TCTP (TCTP siRNA #2) for 72 hr (n=4). **b-e,** NRVMs were transfected with non-targeting siRNA (CTRL siRNA) or siRNA targeting TCTP (TCTP siRNA #2), or BNIP3 (BNIP3 siRNA #2), or a mixture of both (TCTP siRNA #2 & BNIP3 siRNA #2). Expression of TCTP, BNIP3 and GAPDH proteins in NRVMs was analyzed after 72 hr **(b)**. Cell death **(c)** and apoptosis **(d)** were determined by calcein-AM (green) / ethidium homodimer-1 (red) staining and flow cytometry after siRNA transfection for 72 hr (n=4). Scale bar, 200 µm. **e,** mPTP opening was assessed by co-loading with calcein/AM and $CoCl_2$ after siRNA transfection for 24 hr. Loss of green fluorescence is indicative of mPTP opening (n=4). Scale bar, 40 µm. **f**, NRVMs were infected with Ad-LC3-GFP (30 MOI) for 8 hr and then transfected with non-targeting siRNA or siRNA targeting TCTP (TCTP siRNA #1), or Bnip3 (Bnip3 siRNA #1) or a mixture of both (TCTP siRNA #1 & Bnip3 siRNA #1) for 48 hr. Green puncta indicate autophagosomes (n=4). Scale bar, 25 µm. **g,** Expression of TCTP protein was evaluated after 24 hr (n=4). *P<0.05, **P<0.01, ***P<0.001. Unpaired, two-tailed Student's *t* test **(a and g)** or One-way ANOVA followed by Tukey's test **(c-f)**.

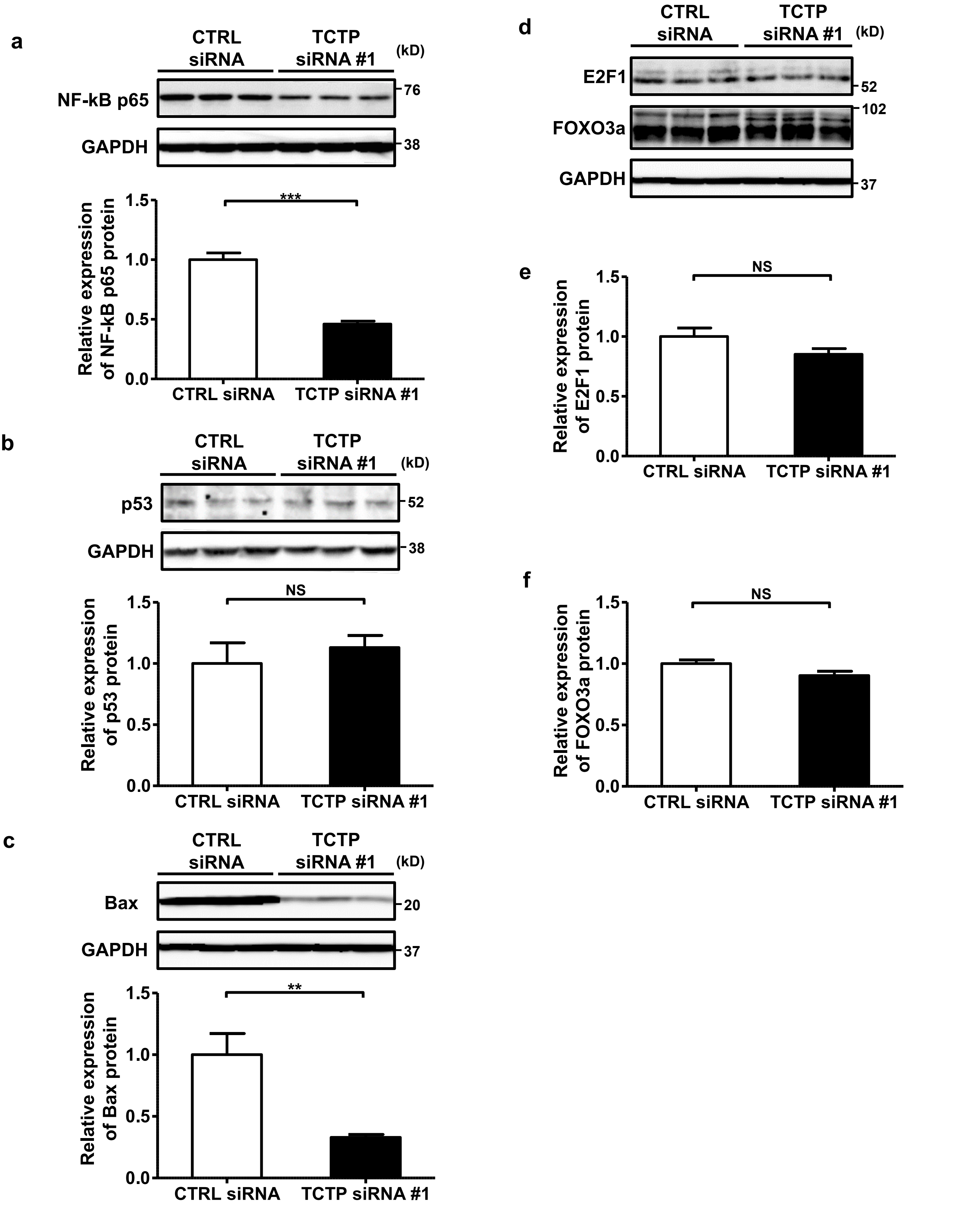

Fig. S4: TCTP down-regulation enhanced NF-kB expression. NRVMs were transfected with non-targeting siRNA (CTRL siRNA) or siRNA targeting TCTP (TCTP siRNA #1). Expression of NF-kB p65 **(a)**, p53 **(b)**, Bax **(c),** E2F1 **(d and e)**, and FOXO3a **(d and f)** proteins in NRVMs was analyzed after 72 hr. n=4. **P*<0.05, ***P*<0.01, ****P*<0.001. Unpaired, two-tailed Student's *t* test.

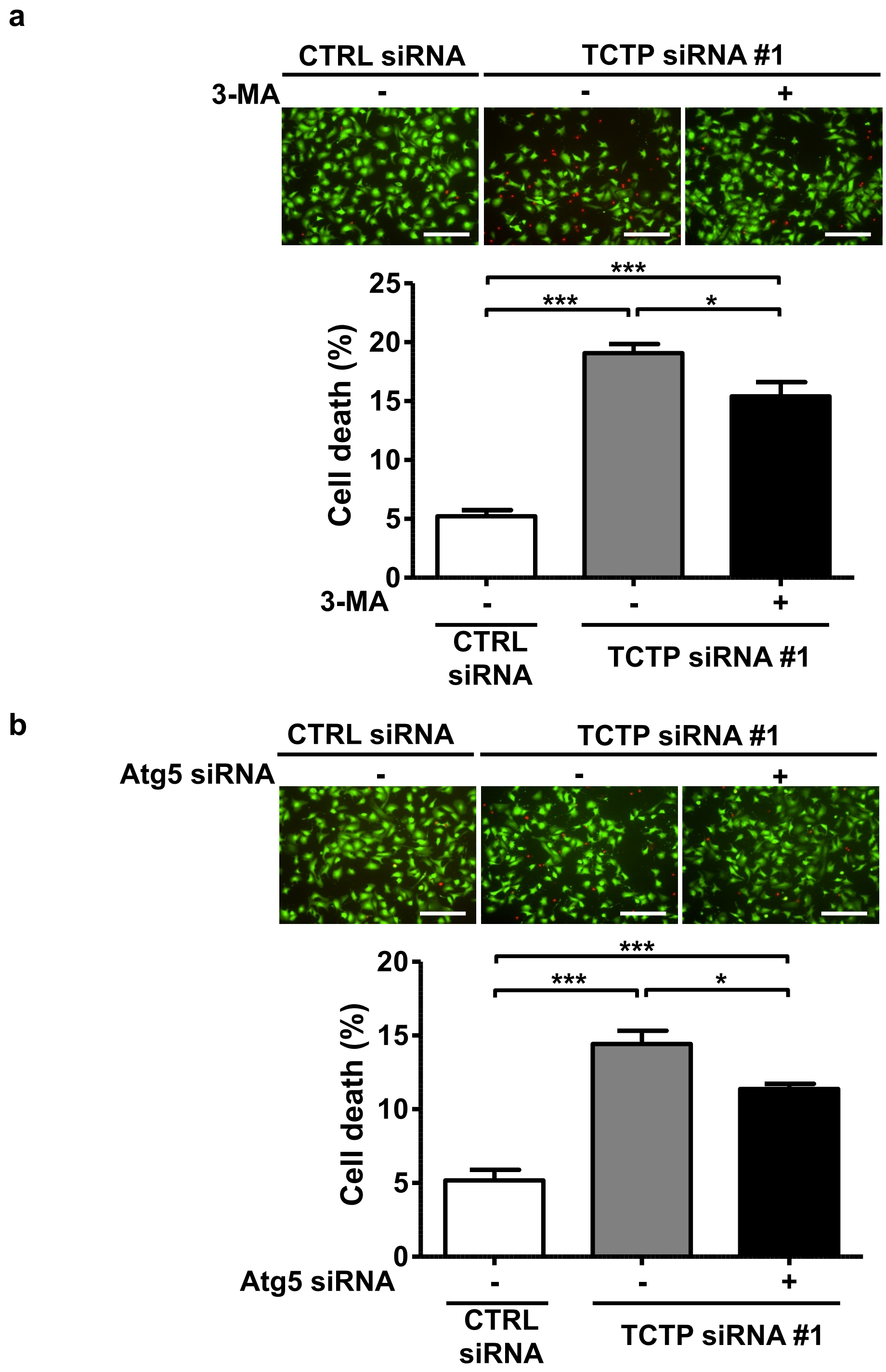

Fig. S5: Inhibition of autophagy suppressed TCTP loss-induced cardiomyocyte death. a, NRVMs were transfected with non-targeting siRNA (CTRL siRNA) or siRNA targeting TCTP (TCTP siRNA #1) and then treated with 3-MA for 24 hr. **b,** NRVMs were transfected with non-targeting siRNA (CTRL siRNA) or siRNA targeting TCTP (TCTP siRNA #1), together with Atg5 siRNA or CTRL siRNA. Cell death was determined by calcein-AM / ethidium homodimer-1 staining after 72 hr (n=4). Live and dead cells were distinguished by calcein-AM (green) and ethidium homodimer-1 (red) staining, respectively. Scale bar, 200 µm. *$P<0.05$, **$P<0.01$, ***$P<0.001$. One-way ANOVA followed by Tukey's test.

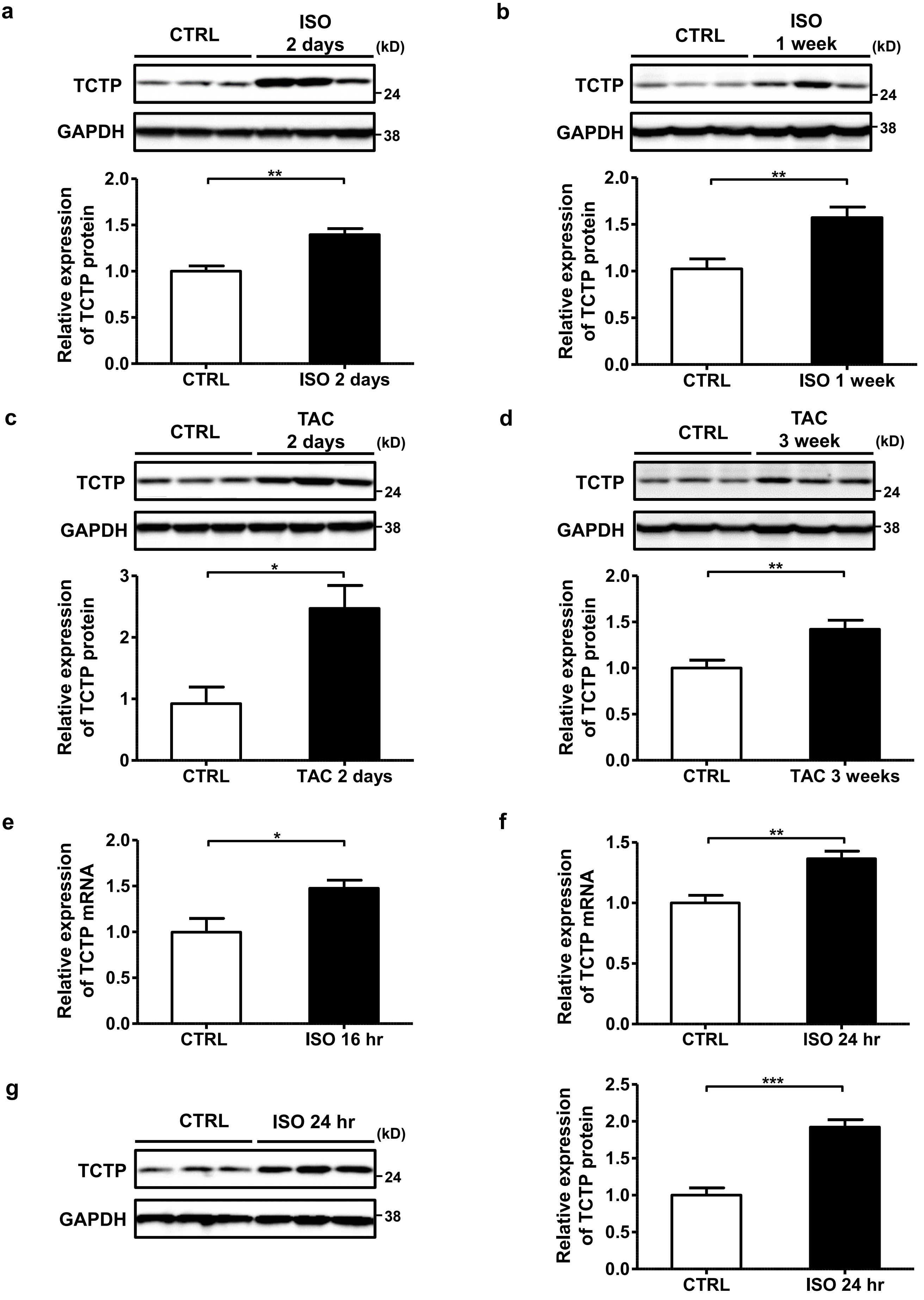

a
CTRL
ISO
2 days
(kD)
TCTP
24
GAPDH
38
Relative expression of TCTP protein
2.0
1.5
1.0
0.5
0.0
**
CTRL
ISO 2 days
b
CTRL
ISO
1 week
(kD)
TCTP
24
GAPDH
38
Relative expression of TCTP protein
**
CTRL
ISO 1 week
c
CTRL
TAC
2 days
(kD)
TCTP
24
GAPDH
38
Relative expression of TCTP protein
3
2
1
0
*
CTRL
TAC 2 days
d
CTRL
TAC
3 week
(kD)
TCTP
24
GAPDH
38
Relative expression of TCTP protein
**
CTRL
TAC 3 weeks
e
Relative expression of TCTP mRNA
*
CTRL
ISO 16 hr
f
Relative expression of TCTP mRNA
**
CTRL
ISO 24 hr
g
CTRL
ISO 24 hr
(kD)
TCTP
24
GAPDH
38
Relative expression of TCTP protein
2.5
***
CTRL
ISO 24 hr

Fig. S6: TCTP expression level in the heart of mice subjected to ISO treatment and TAC. a and b, C57BL/6 mice were treated with ISO (60 mg/kg/day) or normal saline (CTRL) for 2 days **(a)** (n=6) or 1 week **(b)** (n=5~6). **c and d,** C57BL/6 mice received sham operation or TAC for 2 days **(c)** (n=6~7) or 3 weeks **(d)** (n=6~7). Protein expression of TCTP and GAPDH in the heart was analyzed by western blotting. **e and f,** mRNA level of TCTP in NRVMs treated with or without ISO for various times (n=4). **g,** Protein expression of TCTP and GAPDH was evaluated by western blotting (n=4). *$P<0.05$, **$P<0.01$, ***$P<0.001$. Unpaired, two-tailed Student's *t* test.

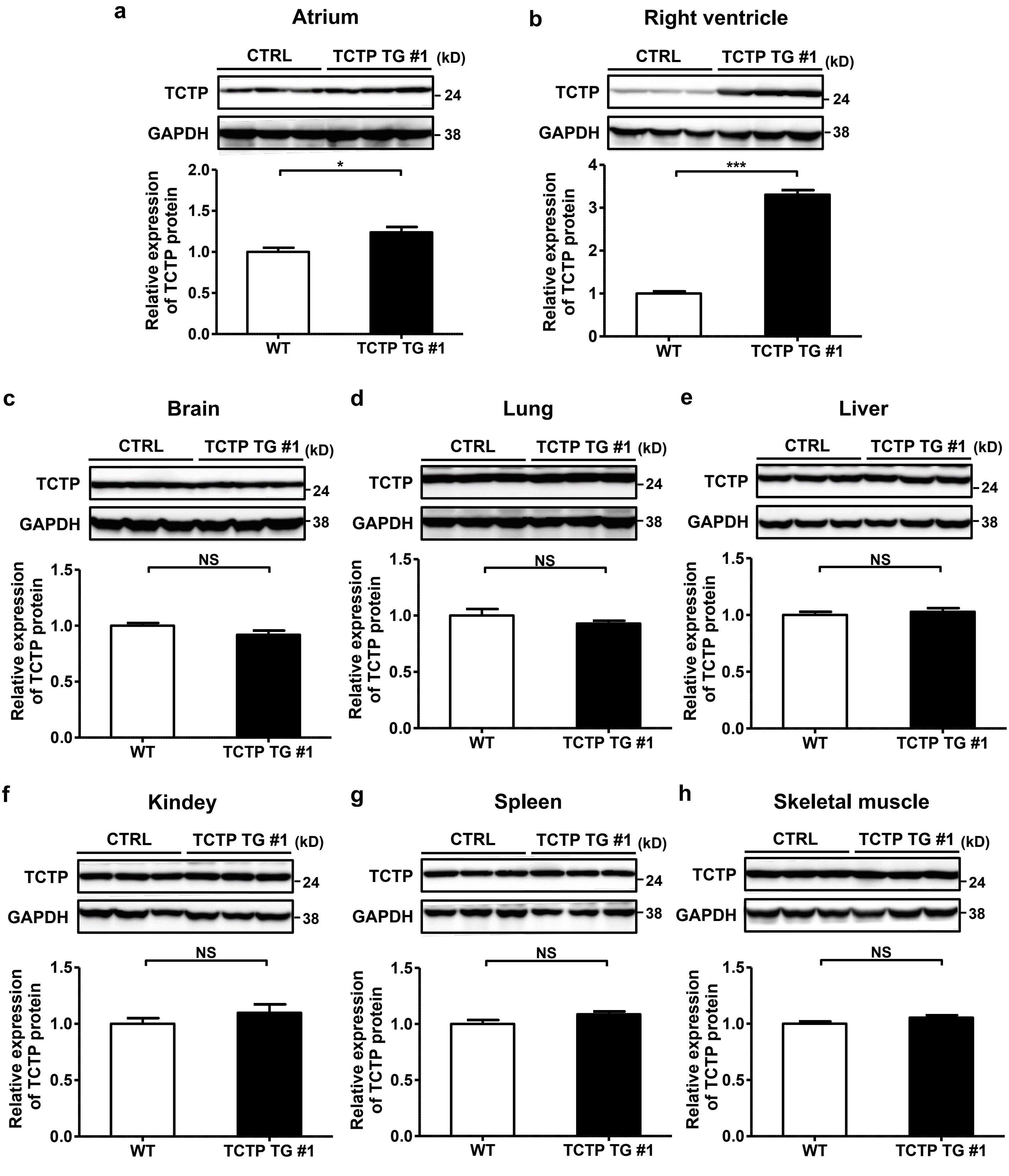

Fig. S7: The expression of TCTP was significant greater in TCTP TG heart, but not in brain, lung, liver, kidney, spleen or skeletal muscle. Protein expression levels of TCTP in atrium **(a)**, right ventricle **(b),** brain **(c)**, lung **(d)**, liver **(e)**, kidney **(f)**, spleen **(g)** and skeletal muscle **(h)** of WT and TCTP TG mice (n=5~6). **P*<0.05, ****P*<0.001. Unpaired, two-tailed Student's *t* test.

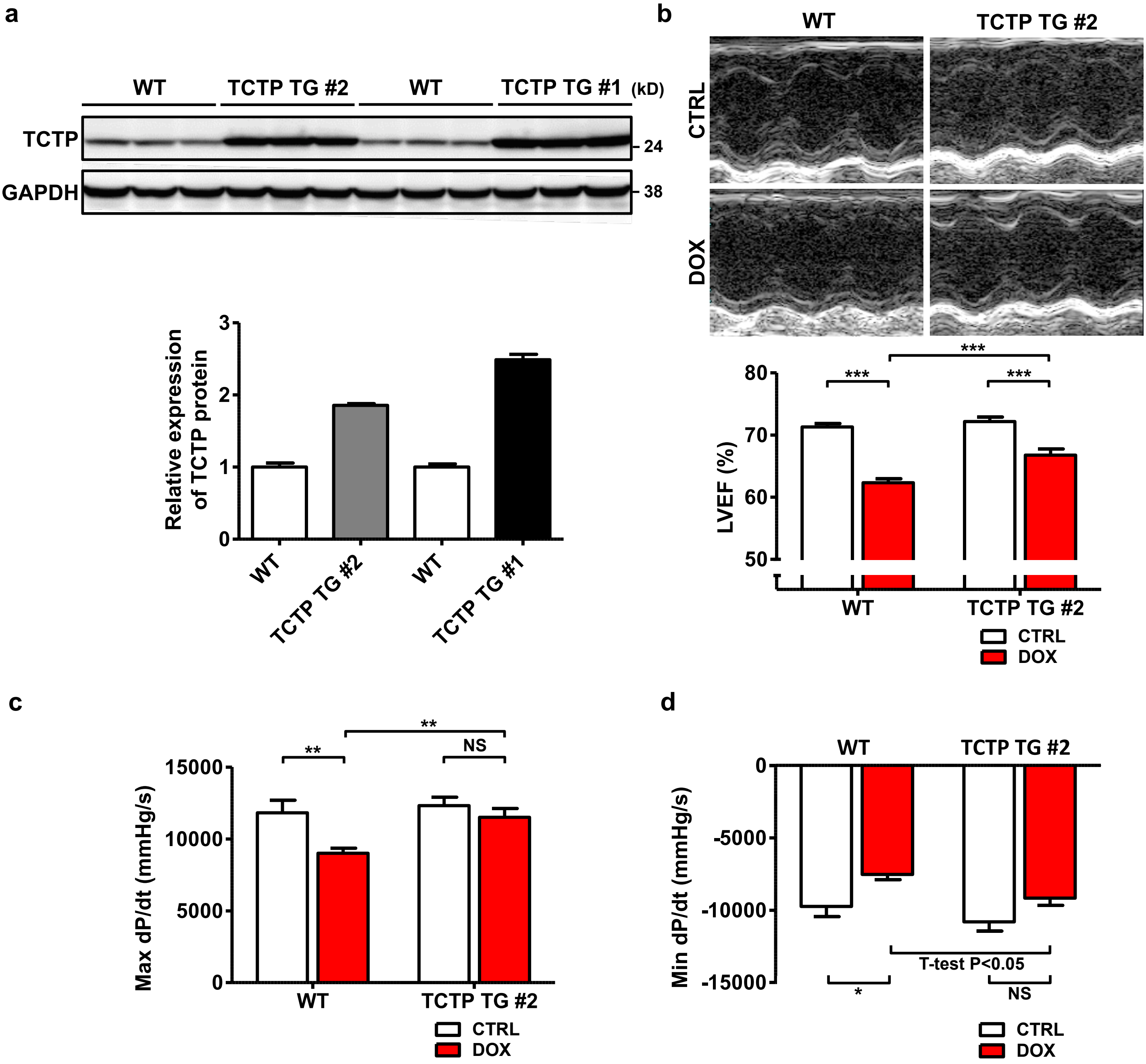

Fig. S8: Cardiomyocyte-specific TCTP overexpression protected mice against DOX-induced cardiac dysfunction. a, TCTP protein expression in left ventricle of WT, TCTP TG #1 and TCTP TG #2 mice (n=3~4). **b-d**, Mice was treated intraperitoneally with DOX (3 mg/kg) or normal saline (CTRL). DOX was administered three times a week, with a total dose of 24 mg/kg. At 5 weeks after the first injection, LVEF was evaluated by echocardiography **(b)** (n=8~14). **c and d,** Max dP/dt and Min dP/dt were evaluated by cardiac catheterization (n=7~11). **P*<0.05, ***P*<0.01, ****P*<0.001. Two-way ANOVA followed by Bonferroni's test.

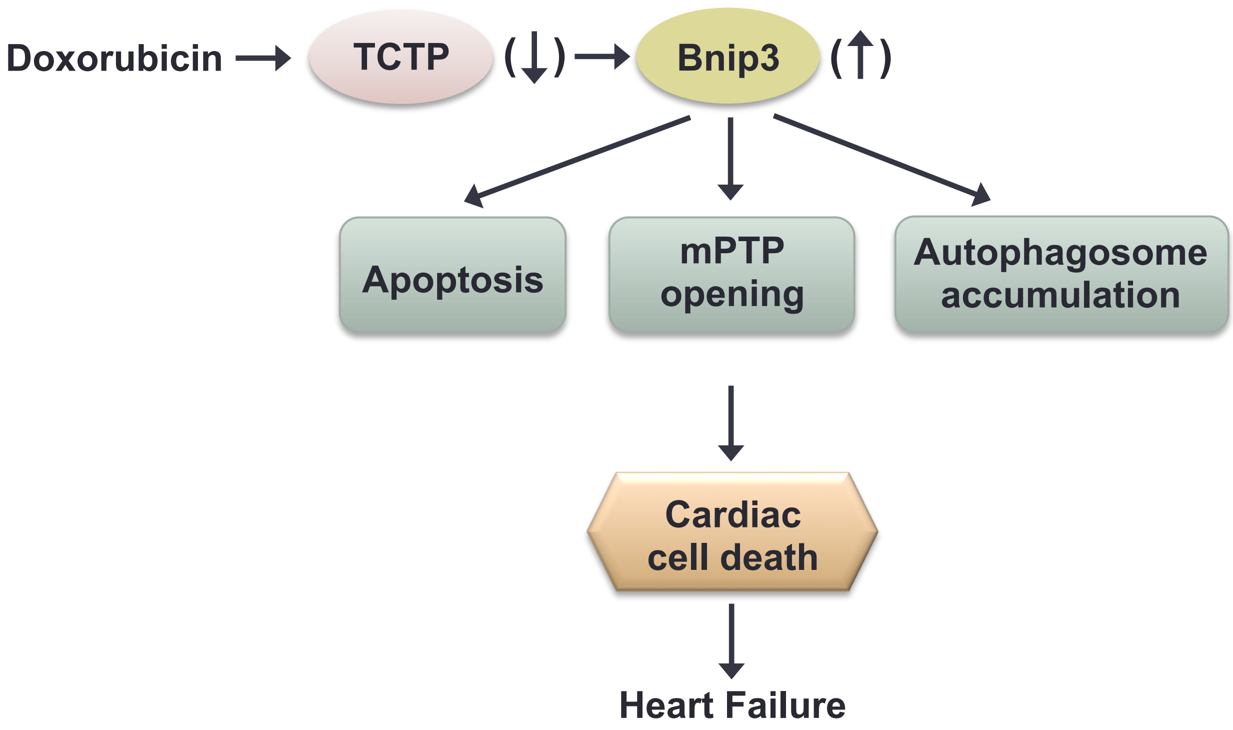

Fig. S9: Schematic model. Mechanisms underlying TCTP loss-induced cardiomyocyte death.

Table S1. siRNA sequences

siRNA Name	Target Sequence
TCTP siRNA#1	UGGUUGCUCUACUGGACUA
TCTP siRNA#2	CTCGCTCCGACTGTCAGGCTA
Bnip3 siRNA#1	D-099769-01 GAAGAGAAGUUGAAAGUAU
	D-099769-02 CAGCUUCCGUCUCUAUUUA
	D-099769-03 AAACUCAGAUUGGAUAUGG
	D-099769-04 UACCAACAGAGCUGAAAUA
Bnip3 siRNA#2	CAGAATAGACTCTTACCACGA
Atg5 siRNA	GCUUUACUCUCUAUCAGGAUGAGAU

Table S2. Comparison of heart weight, body weight and echocardiographic and haemodynamic parameters between the two genotypes

	CTRL		DOX	
	WT	TCTP TG #1	WT	TCTP TG #1
Age (weeks)	8.8±0.66	8.8±0.66	8.0±0.07	8.1±0.06
Body weight (g)	24.8±1.0	24.6±0.8	20.4±0.7 *	19.8±0.5
Heart weight /Body weight (mg/g)	2.82±0.08	2.80±0.09	3.13±0.04	3.19±0.07
EF (%)	71.6±0.66	72.1±0.51	61.9±0.69 *	68.2±0.77 †
FS (%)	34.1±0.53	34.5±0.38	27.6±0.46 *	31.8±0.54 †
Heart rate (b.p.m.)	481±6.4	486±6.7	460±8.0	463±8.0
LVIDd (mm)	3.91±0.04	3.87±0.02	3.98±0.05	3.97±0.03
LVIDs (mm)	2.58±0.04	2.54±0.01	2.88±0.05 *	2.71±0.02 †
Max dP/dt (mmHg/s)	12473±1310	13041±1151	7592±198 *	10581±578 †
Min dP/dt (mmHg/s)	−12332±1184	−11601±591	−6962±431 *	−10191±760 †
Tau (ms)	0.010±0.001	0.011±0.004	0.013±0.001	0.008±0.001

*, P<0.05 WT CTRL group vs. WT DOX proup. †, P<0.05 WT DOX group vs. TCTP TG #1 DOX group

Table S3. Comparison of heart weight, body weight and echocardiographic and haemodynamic parameters between the two genotypes

	CTRL	DHA	
		WT	TCTP TG #1
Age (weeks)	8.6±0.15	8.5±0.15	8.7±0.8
Body weight (g)	23.7±0.47	24.2±0.45	24.9±0.57
Heart weight /Body weight (mg/g)	3.40±0.10	3.33±0.12	3.36±0.11
EF (%)	71.4±0.76	58.9±1.46 *	67.5±1.09 †
FS (%)	34.3±0.54	25.8±0.90 *	31.3±0.75 †
Heart rate (b.p.m.)	466±3.9	448±4.6	466±7.1
LVIDd (mm)	4.05±0.04	4.20±0.09	4.17±0.06
LVIDs (mm)	2.66±0.04	3.12±0.09 *	2.87±0.06 †
Max dP/dt (mmHg/s)	10270±663	7670±374 *	1262±601 †
Min dP/dt (mmHg/s)	−8858±372	−6705±428 *	−8162±418 †
Tau (ms)	0.011±0.001	0.013±0.002	0.010±0.001

*, P<0.05 CTRL group vs. WT DHA proup. †, P<0.05 WT DHA group vs.TCTP TG #1 DHA group

Table S4. Comparison of heart weight, body weight and echocardiographic and haemodynamic parameters between the two genotypes

	CTRL		DOX	
	WT	TCTP TG #2	WT	TCTP TG #2
Age (weeks)	9.5±0.51	9.4±0.37	9.4±0.33	9.6±0.37
Body weight (g)	28.0±0.5	26.2±0.5	20.7±0.5 *	20.2±0.4
Heart weight /Body weight (mg/g)	3.04±0.04	2.97±0.06	3.12±0.06	3.08±0.08
EF (%)	71.3±0.55	72.2±0.75	62.3±0.63 *	66.7±1.01 †
FS (%)	34.0±0.38	34.6±0.59	27.9±0.39 *	30.8±0.65 †
Heart rate (b.p.m.)	492±8.5	495±7.2	462±6.6	478±12.0
LVIDd (mm)	4.05±0.05	4.04±0.07	4.06±0.04	3.92±0.06
LVIDs (mm)	2.68±0.05	2.64±0.06	2.93±0.04 *	2.72±0.06 †
Max dP/dt (mmHg/s)	11821±871	12323±588	9007±346 *	11500±618 †
Min dP/dt (mmHg/s)	−9740±699	−10807±642	−7535±362 *	−9163±504
Tau (ms)	0.009±0.001	0.008±0.001	0.011±0.001	0.008±0.001 †

*, P<0.05 WT CTRL group vs. WT DOX proup. †, P<0.05 WT DOX group vs. TCTP TG #2 DOX group
